# Characterizing malignant prognostic signatures in primary glioma based on single-cell and bulk transcriptome sequencing

**DOI:** 10.1371/journal.pone.0349749

**Published:** 2026-06-05

**Authors:** Yuhui Gong, Haoran Guo, Haolong Ding, Xiaolong Hu, Zhiliang Ding, Mian Ma, Junjie Chen

**Affiliations:** 1 Department of Neurosurgery, Nanjing Medical University Affiliated Suzhou Hospital, Suzhou, Jiangsu Province, China; 2 Central Laboratory, The Affiliate Suqian Hospital of Xuzhou Medical University, Suqian, Jiangsu Province, China; 3 Department of General Surgery, Suzhou Ninth Hospital Affiliated to Soochow University, Suzhou, Jiangsu Province, China; The University of Texas, MD Anderson Cancer Center, UNITED STATES OF AMERICA

## Abstract

Glioma, characterized by its highly invasive nature, presents significant challenges in prognosis and treatment resistance. The advent of single-cell RNA sequencing (scRNA-seq) has facilitated a more nuanced understanding of the cellular and molecular landscapes of glioma cells. In this study, conducting a comprehensive analysis of scRNA-seq and bulk RNA-seq data, employing Cox regression and Least Absolute Shrinkage and Selection Operator (LASSO) methods, we identified three malignant prognostic signatures: IGFBP2, MDK, and RARRES2. The predictive accuracy of this model was validated across both The Cancer Genome Atlas (TCGA) and Chinese Glioma Genome Atlas (CGGA) cohorts. Additionally, we explored the correlation of these signatures with drug responsiveness and immune cell infiltration. Differential expression validation and functional analyses of RARRES2 were performed using external Gene Expression Omnibus (GEO) datasets and in-house samples. In both glioma and pan-cancer contexts, RARRES2 expression is significantly positively correlated with the infiltration of M2-like macrophages, NK cells, and CD8^+^ T cells. Given that RARRES2 receptors are predominantly found in myeloid and glioma cells, we hypothesize that RARRES2 may regulate tumor progression through autocrine pathways and influence macrophage recruitment and differentiation via paracrine pathways. Collectively, our findings provide valuable insights into potential novel prognostic markers for glioma, potentially enhancing the accuracy of prognostic predictions and serving as promising therapeutic targets.

## 1. Introduction

Glioma, the most prevalent primary malignant tumor of the central nervous system, accounts for approximately 80% of all malignant brain tumors and is characterized by high invasiveness and recurrence rates [1]. In clinical practice, the World Health Organization (WHO) classification (WHO Grade) serves as a conventional tool for assessing the risk and treatment requirements of specific glioma patients [2]. While this approach is simple and efficient, it lacks sufficient precision, often failing to meet post-treatment expectations. Within each grade, there are still diverse tumor subtypes with varying biological invasiveness. In 2016, the WHO first integrated molecular markers such as isocitrate dehydrogenase (IDH) mutations, 1p/19q (the short arm of chromosome 1 and the long arm of chromosome 19) co-deletion, and promoter methylation of the MGMT gene into the classification criteria [3]. Glioma patients typically undergo maximal safe resection, followed by postoperative radiotherapy and chemotherapy [4]. Despite recent advancements in targeted therapies, there has been no substantial improvement in the survival rates of glioma patients [5,6]. Over 45% of patients with lower-grade glioma (LGG) experience recurrence within 5 years [7,8], with 17% to 32% progressing to glioblastoma multiforme (GBM) [9–11], and the median survival for GBM patients is only 12–15 months [12,13]. Therefore, identifying new specific prognostic molecular markers and therapeutic targets is crucial for advancing the treatment of glioma.

In recent years, with the advancements in bioinformatics technology, a multitude of prognostic gene signatures have been developed. Multigene signatures have opened new avenues for early identification of high-risk patients, guiding treatment decisions, and improving survival outcomes [14,15]. However, most studies are limited to traditional bulk RNA-seq or bulk RNA-array technologies, or lack large cohort validation. With the continuous development of high-throughput detection technologies and the improvement of open-source databases, for most diseases, sample scarcity and data insufficiency are no longer limitations for clinical research [16]. The advent of single-cell RNA sequencing (scRNA-seq) technology has provided a novel means for studying sample heterogeneity. Compared to traditional bulk transcriptome technologies, which probe average gene expression across cell populations, scRNA-seq can deeply characterize the transcriptional states of individual cells, revealing cellular heterogeneity within samples and describing their molecular features [17].

In this study, we integrated copy number variation (CNV), cell proliferation, and stemness score to identify highly malignant glioma cells from scRNA-seq data. Utilizing differential expression analysis, Cox regression, and LASSO analysis for dimensionality reduction, we developed a three-gene model comprising IGFBP2, MDK, and RARRES2, which demonstrated the best regression efficiency. The malignant prognostic scores (MPS) calculated based on this model effectively predict patient prognosis, and its robustness has been validated in TCGA and CGGA cohorts. These signatures are not only closely related to traditional molecular markers but also significantly enhance the predictive power of these glioma markers.

Furthermore, we conducted additional validation of RARRES2 expression dysregulation and biological functions in glioma and pan-cancer using external validation cohorts and in vitro tumor models. RARRES2 has been implicated in poor outcomes and inflammatory responses activation, and its receptors are primarily located in myeloid cells and malignant cells, highlighting the potential importance of RARRES2 in the recruitment of immune cells and cancer progression.

## 2. Methods

### 2.1. Data source

ScRNA-seq data were obtained from the Gene Expression Omnibus (GEO) database under accession number GSE135045, comprising 7 GBM samples. Glioma bulk RNA-seq data and corresponding clinical features were retrieved from The Cancer Genome Atlas (TCGA), the Chinese Glioma Genome Atlas (CGGA) and GEO database. Formalin-fixed paraffin-embedded samples from TCGA were excluded due to their inherent limitations in sequencing analyses [18,19]. Normal tissue bulk RNA-seq data were retrieved from Genotype-Tissue Expression (GTEx) and Human Protein Atlas (HPA). The TCGA and GTEx combined cohort was downloaded from the UCSC Xena. The summary of genomic alteration frequency was downloaded from cBioPortal. The RNA expression microarray datasets GSE74187, GSE107850, GSE147352 and GSE16011 were retrieved from the GEO database for expression validation.

### 2.2. Single-cell transcriptome sequencing data analysis

The R package “Seurat” (version 4.3.0) [20] was utilized to process the scRNA-seq. This involved conducting data quality control, normalization, scaling, identifying hypervariable genes, performing principal component analysis (PCA), and applying uniform manifold approximation and projection (UMAP). Initially, the CreateSeuratObject function was employed to construct a Seurat object, and low-quality cells were filtered out based on the following criteria: (1) cells with fewer than 500 unique molecular identifiers (UMI) or more than 8000 UMI; (2) cells with more than 20% mitochondrial UMI counts; and (3) cells with more than 50% ribosomal UMI. Subsequently, the ‘LogNormalize’ method of the NormalizeData function was applied to normalize the data with a scaling factor of 10,000. The FindVariableFeatures function was then utilized to identify 2000 highly variable genes for further analysis. The RunPCA function was employed for data dimensionality reduction, selecting the first 30 principal components for downstream analysis. Ultimately, the UMAP algorithm was used to visualize the dimensionality reduction of cells.

Batch effects were adjusted using the “harmony” package (version 0.1.1) [21]. Cell types were initially annotated by manual curation using canonical marker genes (see S1 Table). To validate the manual annotations, we applied automated reference-based annotation using SingleR (version 2.4.1) with default parameters and the “HumanPrimaryCellAtlasData” reference. In addition, we compared our cell type proportions with published GBM single-cell atlases [22] to ensure consistency. The CellCycleScoring function in Seurat was employed to calculate the cell cycle score of each cell. Proliferation and stemness module scores were computed using the AddModuleScore function [23]. Copy number variations (CNVs) were estimated using the “infercnv” package (version 1.10.1) [24], using T cells as a reference. HLA genes on chromosome 6 were excluded due to their potential to exhibit CNVs in immune cells [25].

### 2.3. Differential expression analysis

Differential expression analysis was performed using the “DESeq2” package (version 1.34.0) [26]. Genes were retained if they exhibited non-zero expression in more than 80% of samples and had an average raw count greater than 1. Genes meeting the criteria of an absolute log2 fold change of 0.585 or greater and an adjusted p-value less than 0.05 were considered differentially expressed genes.

### 2.4. Identification of malignant prognostic signature

Firstly, univariate Cox regression analysis was conducted to identify significant prognostic genes. Subsequently, the Least Absolute Shrinkage and Selection Operator (LASSO) regression was applied to further reduce the number of candidate genes and construct the optimal prognostic signature. The “glmnet” package (version 4.1.7) [27] was utilized to determine the optimal penalty parameter (lambda.min) through 1000 iterations of 10-fold cross-validation. Patient-specific risk scores were calculated by integrating the coefficients and expression levels of the prognostic genes, as follows: ∑(Coef_i_ × Expr_i_), where Coef_i_ denotes the coefficient of gene i, and Expr_i_ represents the expression level of gene i.

To evaluate the discrimination ability, Kaplan–Meier analysis and the concordance index (C-index) were performed using the “survival” package (version 3.2.11). Subsequently, calibration curves were generated to assess the agreement between predicted and observed outcomes using the “rms” (version 8.1.0) package, and time-dependent receiver operating characteristic (ROC) curve analysis was performed using the “timeROC” package (version 0.4) [28]. Samples with survival times exceeding 10 years were excluded.

### 2.5. Cell culture and mouse model establishment

GL261 cells were cultured in DMEM medium (containing 10% FBS and penicillin/streptomycin). The cells were cultured in a humidified atmosphere containing 5% CO_2_ at 37°C.

All experimental procedures were approved by the Institutional Animal Care and Use Committee of the Animal Experimental Center at Soochow University. Animal breeding was managed by specialized personnel from the animal center. This study was conducted in strict accordance with the recommendations of the guide for the care and use of laboratory animals issued by the Ministry of Science and Technology of China.

Five female C57BL/6J mice, weighing 18 ± 2g and aged 3–4 weeks, were purchased from Changzhou Cavens Experimental Animal Co., Ltd. An intracranial GBM mouse model was established through stereotactical inoculation of GL261 cells (200,000 cells per mouse in 2 μL of serum-free media) into the brain (1.8 mm lateral and 0.6 mm anterior to the bregma; 2 mm of depth) of the mice that were anesthetized using 1% pentobarbital sodium. The tumor size was evaluated weekly through the animal imaging system. Daily monitoring included activity, weight loss (>20%), and distress signs. Veterinarian supervised endpoint determinations. Housing: All mice were housed in the animal laboratory of Soochow University (temperature 20 ~ 24 ℃, 40% ~ 45% humidity, and a 12 h light-dark cycle), and the mice were free to eat and drink during the rearing period. All mice were euthanized on day 28 after inoculation or reached Tumor Volume Endpoint: Mice were euthanized when tumor burden reached 1000 mm³ (calculated as 0.5 × length × width²), and complete excision of brains was performed, followed by isolation of the tumor from each mouse.

Mice were deeply anesthetized via intraperitoneal injection of 1% pentobarbital sodium. Adequate anesthetic depth was confirmed by the absence of the pedal withdrawal reflex. After achieving full anesthesia, mice were humanely euthanized by cervical dislocation. Subcutaneous tumors were then immediately dissected and harvested under aseptic conditions. As tumor collection was performed as a terminal procedure following euthanasia, no additional analgesics were required. All procedures were carried out rapidly to minimize discomfort, and every effort was made to alleviate animal suffering during handling and experimentation. All animal protocols were approved by the Institutional Animal Care and Use Committee (IACUC) and performed in accordance with institutional guidelines for the care and use of laboratory animals.

After intracranial injection of GL261 cells and tumor development, glioma tissues along with adjacent normal brain tissues were harvested from the mice for Western blot.

### 2.6. Western blotting

The harvested glioma tissues and adjacent normal mouse brain tissues were lysed and subsequently used for Western blot analysis. Separate total proteins using SDS-PAGE (Sodium Dodecyl Sulfate Polyacrylamide Gel Electrophoresis), transfer to the nitrocellulose membrane, block with 5% skimmed milk, and sequentially incubate with primary antibodies and secondary antibodies coupled with horseradish peroxidase (HRP). Visualize using chemiluminescence. The primary antibodies used for protein immunoblotting are as follows: RARRES2/Chemerin (10216–1-AP, Proteintech, 1:2000); Tubulin (66031–1-Ig, Proteintech, 1:2000); HRP-conjugated Goat Anti-Rabbit IgG(H + L) (SA00001–2, Proteintech, 1:2000).

### 2.7. Immunohistochemical analysis

Tumor tissues were obtained from the patients who underwent curative resection from 01/03/2022 to 01/03/2024 at Department of Neurosurgery, Nanjing medical university affiliated Suzhou Municipal Hospital. The study was conducted in accordance with the ethical principles stated in the Declaration of Helsinki and approved by the Bio-medical Research Ethics Committee of Nanjing Medical University Affiliated Suzhou Hospital (No.268(2024)). All of the patients provided written informed consent. This study utilized archived paraffin-embedded glioma tissue samples from Suzhou Municipal Hospital under approval on 28/04/2025 by Suzhou Municipal Hospital Ethics Committee. Written informed consent was obtained from all participants prior to tissue collection, including permission for future research use of anonymized data. Data accessed included [transcriptome sequencing, IHC results]. No additional clinical records beyond pathological diagnosis were used. All glioma tissue samples were processed under strict de-identification protocols: Patient IDs were replaced with anonymized codes; Sample metadata excluded name, ID, address, and exact diagnosis dates. Dewaxing of paraffin sections to water involves sequentially immersing the sections in an environmentally friendly dewaxing solution, followed by anhydrous ethanol, and then rinsing with distilled water. Sections should be heated in citrate buffer (pH = 6.0) after being submerged in 3% hydrogen peroxide (H_2_O_2_) for 15 min in order to retrieve the antigen. Allow the slides to cool naturally and then rinse them in PBS for three washes. For twenty-five minutes, submerge the parts in a 3% hydrogen peroxide solution at room temperature without exposure to light. Then, place the slides in PBS for 3 washes. Add 3% bovine serum albumin (BSA) evenly to cover the tissue within the histology ring. Drop the pre-diluted primary antibody onto the slide and incubated overnight at 4 °C. Rinse the slides three times in PBS. After air-drying the sections, add the secondary antibody corresponding to the species of the primary antibody, covering the tissue, and incubate at room temperature for 50 min. Rinse the slides three times in PBS. After drying the sections, add freshly prepared DAB coloration. Restain with hematoxylin for about 3 min, followed by rinsing with tap water. Finally, dehydration, gum sealing, and microscopic photography were carried out. The proportion of positive cells was determined by counting at least 1,000 cells in three random microscopic fields for each sample. The following primary antibodies were employed for immunohistochemical staining: RARRES2/Chemerin (10216–1-AP, Proteintech, 1:200).

### 2.8. Quantitative real‐time reverse transcription PCR (qRT‐PCR)

Total RNA was extracted using TRizol Reagent and 5 ug of RNA was used to synthesize cDNA according to the manufacturer′s instructions. The PCR conditions consisted of 5 min at 95 °C 1 cycle，30 s at 95 °C，30 s at 55 °C，30 s at 72 °C and 7 min at 72 °C 40 cycles. The sequences of primers are as follows:

RARRES2-F: TTGCTGATCTCCCTAGCCCTA;

RARRES2-R: TGGGTGTTTGTGGAACTCCTC;

IGFBP2-F: CAGACGCTACGCTGCTATCC;

IGFBP2-R: CCCTCAGAGTGGTCGTCATCA;

MDK-F: CAAGGGACCCTGAAGAAGGC;

MDK-R: CTTTGGTCTTTGACTTGCTCTTGG;

ACTIN-F: TGACTTCAACAGCGACACCCA;

ACTIN-R: CACCCTGTTGCTGTAGCCAAA.

The relative fold changes in mRNA expression were calculated using the 2^–ΔΔCT^ method, where the average of ΔCT values for the amplicon of interest was normalized to that of Actin.

### 2.9. Gene set enrichment analysis and gene set variation analysis

Gene sets of the Kyoto Encyclopedia of Genes and Genomes (KEGG), Gene Ontology Biological Process (GOBP), and HALLMARK (version 2023.2) were retrieved from the Molecular Signatures Database (MSigDB) [29]. Gene Set Enrichment Analysis (GSEA) was performed using the R package “clusterProfiler” (version 4.9.0.2) [30] to identify potential differences in biological pathway activity. Gene set variation analysis (GSVA) was performed using the “gsva” function of the GSVA package (version 1.44.5) [31].

### 2.10. Prediction of chemosensitivity

The prediction of the half-maximal inhibitory concentration (IC50) was conducted using the “oncoPredict” package [32], with GDSC v2 data serving as the training set. A lower IC50 value indicates greater sensitivity to the drug in cancer treatment. Consistent trends across four bulk RNA-seq cohorts (TCGA-GBM, TCGA-LGG, CGGA-mRNAseq365, and CGGA-mRNAseq693) were visualized in a dot plot.

### 2.11. Immune cell infiltration analysis

The ESTIMATE algorithm was harnessed to derive Immune Scores (version 1.0.13) [33]. Single-sample Gene Set Enrichment Analysis (ssGSEA) and CIBERSORT were employed to quantify immune cell infiltration levels in the glioma cohort [34]. Additionally, immune cell infiltration data from multiple algorithms, including TIMER, quanTIseq, MCP-counter, xCell, and EPIC, were retrieved from the Tumor IMmune Estimation Resource (TIMER) 2.0 database [35].

### 2.12. Statistical analysis

All data processing, statistical analyses, and plotting were performed using R 4.2.0 software. Spearman correlation was used to assess relationships between continuous variables. For comparing two groups, the statistical significance of variables was determined using the Wilcoxon rank-sum test. Meta analysis were performed using the “meta” package (version 8.0.2). All statistical tests were two-sided, with *p* < 0.05 considered statistically significant.

## 3. Results

### 3.1. Single-cell transcriptional profiling of glioma

**Fig 1:**
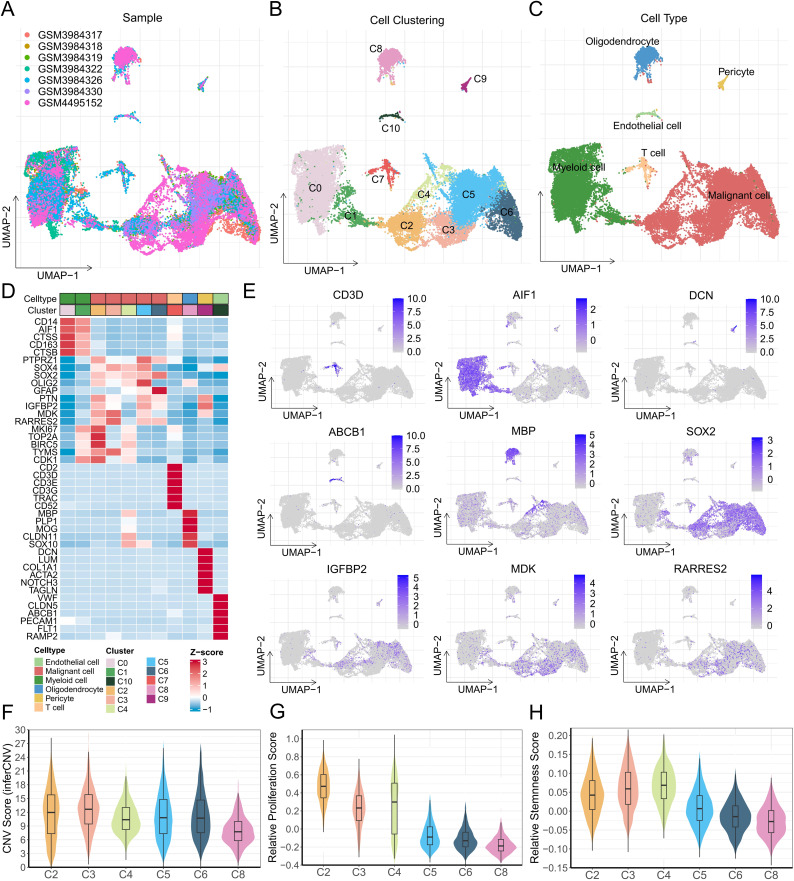
Single-cell atlas of glioma tissue. (A-C) UMAP projection of the cellular landscape colored by sample source (A), clusters (B), and cell types (C). (D) Heatmap displaying the mean expression levels of marker genes across clusters. (E) UMAP plots showing the expression of classical molecular markers. (F-H) Boxplots comparing the CNV score (F), proliferation score (G), and stemness score (H) across clusters.

A total of 27,642 cells from 7 GBM samples passed quality control filtering, and batch effects were effectively corrected using the Harmony method (Fig 1A). At a resolution of 0.3, cells were stratified into 11 clusters (C0–C10) (Fig 1B). Immune cell signature genes (CD45, CD3D, AIF1) were specifically enriched in clusters C0, C1, and C7, whereas stromal markers (PECAM1, DCN, LUM) were predominantly expressed in clusters C9 and C10 (Figs 1D, E and S1A). Compared with cluster C8, clusters C2–C6 exhibited higher copy number variation (CNV) scores within non-immune and non-stromal cell populations (Fig 1F), along with elevated proliferation and stemness scores (Fig 1G, H). These clusters also showed increased G2/M phase scores and a reduced G1 phase ratio relative to oligodendrocytes (C8), indicating active cell cycle progression (S1B–D Fig). GSVA further revealed that clusters C2-C6 were significantly enriched in pathways related to cell cycle progression and oncogenic signaling, including MYC, and E2F (S1E Fig). Consistently, known GBM tumor signature genes (SOX2, SOX4, and OLIG2) [36] were markedly upregulated in these clusters, whereas cluster C8 specifically expressed oligodendrocyte markers (MBP, PLP1, MOG) (Fig 1D, E, S2 Table). Consequently, based on marker gene expression patterns and the above features, all clusters were annotated into six major cell types: myeloid cells (C0 and C1), T cells (C7), pericytes (C9), endothelial cells (C10), oligodendrocytes (C8), and malignant cells (C2–C6) (Fig 1B, C).

### 3.2. Identification of malignant prognostic signatures

**Fig 2:**
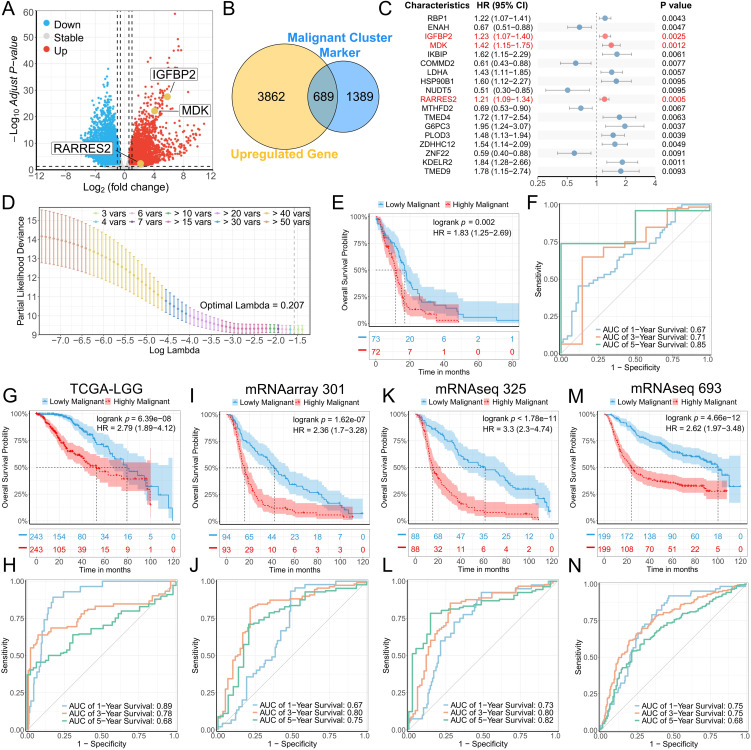
Construction and validation of the prognostic model. (A) Volcano plot of differential analysis between GBM and normal tissues in the TCGA-GBM cohort. (B) Venn diagram of upregulated genes in GBM and malignant cluster marker genes. (C) Forest plot showing the prognostic effect on OS based on univariate Cox regression in the TCGA-GBM cohort. (D) Cross-validation of the prognostic effect using LASSO regression. (E) Kaplan-Meier survival curves comparing OS between low- and high-MPS groups in the TCGA-GBM cohort. (F) Time-dependent ROC analysis assessing the malignant score for predicting OS at 1, 3, and 5 years in the TCGA-GBM cohort. (G, I, K, M) Kaplan-Meier survival curves comparing OS between low- and high-MPS groups in the TCGA-LGG (G), mRNAarray 301 (I), mRNAseq 325 (K), and mRNAseq 639 (M) cohorts. (H, J, L, N) Time-dependent ROC analysis assessing the malignant score for predicting OS at 1, 3, and 5 years in the TCGA-LGG (H), mRNAarray 301 (J), mRNAseq 325 (L), and mRNAseq 639 (N) cohorts.

Considering the higher CNV, stemness, and proliferation scores observed in clusters 2, 3, and 4, these clusters were designated as representative malignant cells, and differential analysis was performed to identify cluster-specific marker genes. Among these, 689 genes were also upregulated in GBM samples compared with normal controls in the TCGA-GBM cohort (Fig 2A, B). To construct malignant prognostic signatures for glioma patients, univariate Cox regression analysis was performed on these malignant DEGs in the discovery cohort (TCGA-GBM), and a total of 55 genes met the cutoff of *p* < 0.05, with genes exhibiting *p* < 0.01 displayed in a forest plot (Fig 2C). These genes were subsequently subjected to LASSO-Cox proportional hazards regression with 10-fold cross-validation. The LASSO coefficient profile plotted against the log(λ) sequence identified three optimal coefficients using the minimum λ criterion (Fig 2D). Ultimately, a three-gene signature comprising IGFBP2, MDK, and RARRES2 demonstrated optimal predictive performance (Fig 2D), and malignant prognostic scores (MPS) for each patient were calculated based on the LASSO model. Survival analysis indicated that patients with lower MPS had significantly prolonged overall survival (Fig 2E, log-rank test, *p* = 0.002). Consistently, ROC curve analysis demonstrated the prognostic performance of the MPS, with 1-, 3-, and 5-year AUC values of 0.67, 0.71, and 0.85 in the TCGA-GBM cohort (Fig 2F).

### 3.3. External validation of MPS prediction efficiency

To confirm the robustness and efficacy of the MPS across diverse populations, the same calculation was applied to four external validation cohorts from the TCGA and CGGA databases (TCGA-LGG, mRNAarray 301, mRNAseq 325, and mRNAseq 693). In all cohorts, the median overall survival of patients in the high-MPS group was significantly shorter (Fig 2G, I, K, and M). ROC curve analysis showed that the AUC values for predicting 3-year survival were all above 0.75, while the AUC values for 1- and 5-year survival were all above 0.65 (Fig 2H, J, L, and N). In addition, calibration plots showed strong agreement between predicted probabilities and observed outcomes (S2 Fig), with an average C-index greater than 0.7, indicating good predictive accuracy of the model. These findings collectively demonstrate the robust prognostic performance of the MPS.

To further evaluate whether the malignant prognostic signature (MPS) provides prognostic information independent of established clinical and molecular factors, multivariable Cox regression analyses were performed adjusting for age, WHO grade, IDH mutation status, 1p/19q codeletion, and MGMT promoter methylation. After z-score normalization, MPS remained a statistically significant independent predictor of overall survival in the TCGA cohorts (TCGA-GBM: HR = 1.327, *p* = 0.045; TCGA-LGG: HR = 1.663, *p* = 0.002; S3 Table). In the CGGA cohorts, MPS showed a consistent direction of effect (HR > 1), although statistical significance was not uniformly retained. An additional multivariable model including only molecular covariates (IDH mutation, 1p/19q codeletion, and MGMT promoter methylation) was further constructed, in which MPS remained statistically significant across all five cohorts (S4 Table).

### 3.4. Expression dysregulation and prognostic impact of MPS genes

**Fig 3:**
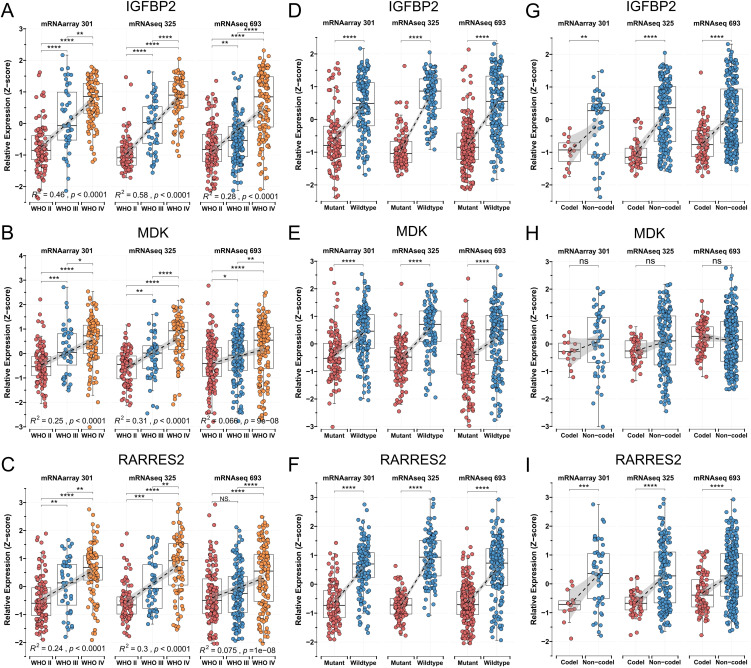
Expression distribution of MPS genes across clinicopathological characteristics. (A-C) IGFBP2 (A), MDK (B), and RARRES2 (C) expression levels are significantly increased in higher-grade gliomas in the CGGA database. (D-F) IGFBP2 (D), MDK (E), and RARRES2 (F) expression levels are significantly increased in gliomas without IDH mutation in the CGGA database. (G-I) Expression distribution of IGFBP2 (G), MDK (H), and RARRES2 (I) across different 1p/19q status groups in three CGGA cohorts.

Next, to further evaluate the role of each MPS gene, differential expression and survival analyses were conducted for the three MPS genes in TCGA and CGGA glioma cohorts. In all three CGGA cohorts, expression levels of all three genes increased significantly with higher tumor grade (Fig 3A–C). Compared to IDH wild-type samples, IDH-mutant samples exhibited significantly lower expression of IGFBP2, MDK, and RARRES2 (Fig 3D–F). Additionally, IGFBP2 and RARRES2 expression were reduced in samples with 1p/19q codeletion (Fig 3G–I). Because IDH mutation and 1p/19q codeletion are well-known markers of prolonged survival and delayed malignant progression [37,38], these expression patterns further highlight the relevance of the three MPS genes to glioma progression.

**Fig 4:**
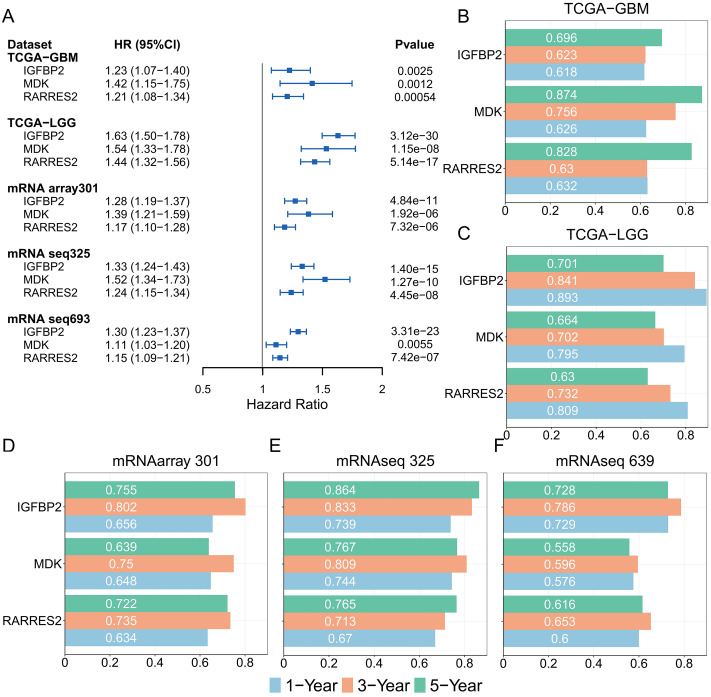
Prognostic impact of MPS genes. (A) Univariate Cox regression analysis of IGFBP2, MDK, and RARRES2 in the TCGA-GBM, TCGA-LGG, mRNAarray 301, mRNAseq 325, and mRNAseq 639 cohorts. (B-F) Time-dependent ROC analysis assessing IGFBP2, MDK, and RARRES2 in the TCGA-GBM (B), TCGA-LGG (C), mRNAarray 301 (D), mRNAseq 325 (E), and mRNAseq 639 (F) cohorts.

Moreover, in both the TCGA and CGGA glioma cohorts, IGFBP2, MDK, and RARRES2 were identified as significant prognostic risk factors (Fig 4A). The AUC values for RARRES2 in predicting 1-, 3-, and 5-year survival all exceeded 0.6, and the average AUC values for IGFBP2 in predicting 1-, 3-, and 5-year survival were 0.73, 0.78, and 0.75, respectively (Fig 4B–F). MDK also demonstrated robust predictive performance across at least four cohorts. Decision curve analysis (DCA) indicated that combining tumor grade with IGFBP2, MDK, or RARRES2 improved prognostic accuracy (S3 Fig). Together, these findings suggest that IGFBP2, MDK, and RARRES2 can serve as effective complements to traditional clinical indicators in glioma prognosis.

### 3.5. Efficacy of MPS genes in predicting drug sensitivity

To explore the relationship between the three prognostic signatures and drug sensitivity, the half-maximal inhibitory concentration (IC50) values of each drug for glioma samples in four sequencing cohorts were calculated. The landscape of correlation and significance between drug sensitivities and malignant signature expression is depicted in S4 Fig.

Positive correlations were observed between the IC50 values of entinostat and the expression of IGFBP2 and MDK. Additionally, the IC50 values of doramapimod, gefitinib, erlotinib, LY2109761, AZD1208, SB505124, vorinostat, and afatinib were positively correlated, while the sensitivities of MG-132, SCH772984, PLX-4720, PD0325901, AZD5582, trametinib, and AZD1332 exhibited negative correlations with IGFBP2 expression. The IC50 values of camptothecin, luminespib, MG-132, irinotecan, 5-fluorouracil, SCH772984, talazoparib, topotecan, rapamycin, and epirubicin were found to be negatively correlated with MDK expression. Furthermore, three consistent pairs of correlations between drug sensitivity and RARRES2 expression, including PCI − 34051, PRIMA−1MET, and venetoclax, were observed across the four cohorts. These findings suggest the potential existence of additional FDA-approved drugs that may hold promise for the treatment of glioma.

### 3.6. Function analysis and expression verification of RARRES2

**Fig 5:**
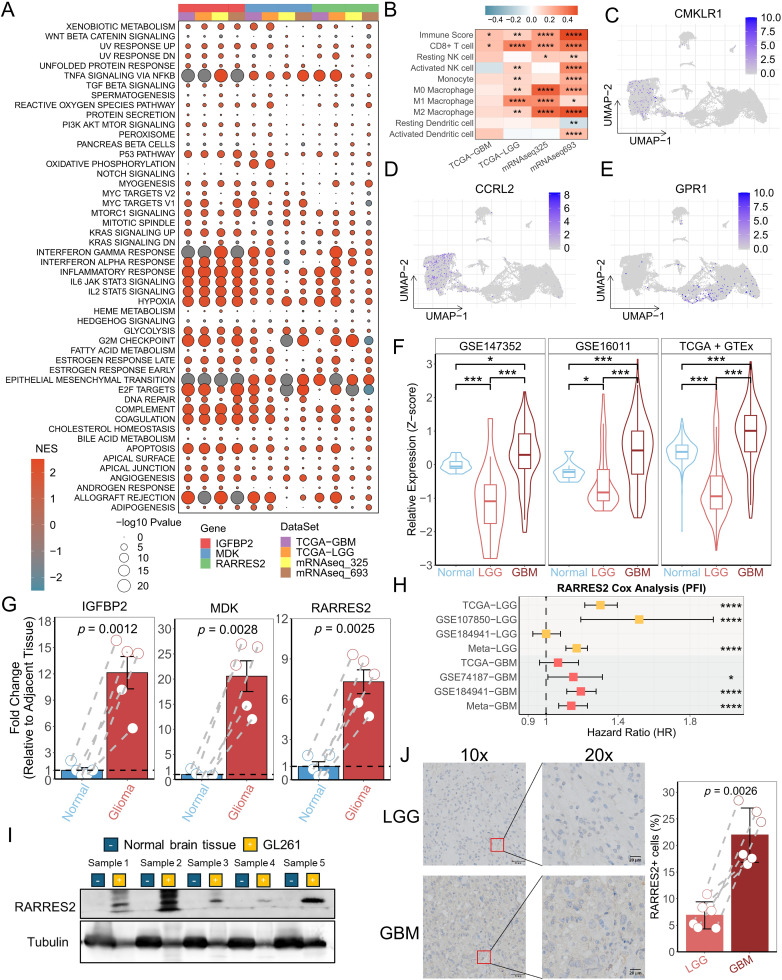
Functional analysis of RARRES2. (A) Hallmark gene set enrichment analysis between high- and low-expression groups of IGFBP2, MDK, and RARRES2 in the TCGA-GBM, TCGA-LGG, mRNAseq 325, and mRNAseq 639 cohorts. Circle size represents the FDR value of the enriched term in each cancer, and color indicates the normalized enrichment score (NES). (B) Correlation analysis between RARRES2 expression levels and immune cell infiltration levels. (C-E) UMAP plots showing the expression distribution of RARRES2 receptors: CMKLR1, GPR1, and CCRL2. (F) qPCR validation of RARRES2 in paired glioma and normal tissues. (G) Expression distribution of RARRES2 in normal brain and glioma tissues in three independent GEO cohorts. (H) Forest plot of meta-analysis for RARRES2 based on univariate Cox regression of PFS. (I) RARRES2 expression levels in paired glioma and adjacent normal tissues detected by Western blot. (J) Representative IHC images and the percentage of RARRES2-positive cells in glioma samples. Scale bars: 50 μm (left) and 20 μm (right). LGG samples, n = 6; GBM samples, n = 6.

To further explore the biological basis of the prognostic marker, GSEA was performed between the high- and low-expression groups. This analysis revealed that multiple immune-related pathways were enriched in the high-expression groups, including IL6-JAK-STAT3 signaling, IL2-STAT5 signaling, interferon-α response, inflammatory response, and the complement system (Fig 5A). Among these, RARRES2 expression showed a consistent positive correlation with the immune score across four cohorts (Figs 5B and S5). Furthermore, using seven different deconvolution algorithms from TIMER 2.0, we analyzed correlations between specific immune cell abundances and gene expression levels. Significant correlations were identified between RARRES2 expression and the abundance of CD8 ⁺ T cells, NK cells, and especially M2 macrophages (Figs 5B and S6). Cross-algorithm analysis indicated that estimates for CD8 ⁺ T cells, monocytes, and macrophages were strongly correlated across methods, supporting the robustness of their association with RARRES2 (S7 Fig). GSEA based on immune cell signatures showed that microglia/macrophage (both M1 and M2 subtypes) were consistently enriched in the high-RARRES2 groups (S8A–B Fig). Notably, M2 macrophages constituted the largest proportion among macrophages and were the primary contributors to changes in macrophage infiltration (S8C–F Fig). Using the square root of fold change × proportion as a relevance metric [39], we found that M2 macrophages, rather than M1 macrophages, were more closely associated with RARRES2 expression (S8G–J Fig).

Previous studies have identified CMKLR1, GPR1, and CCRL2 as primary receptors of RARRES2 [40]. CMKLR1 and CCRL2 were predominantly expressed in myeloid cells, while GPR1 was mainly expressed in malignant cells (Fig 5C–E). In the GTEx-TCGA, GSE16011, and GSE147352 cohorts, RARRES2 expression levels were significantly higher in GBM than in normal brain tissue (Fig 5F). Similarly, in our in-house cohort, both transcriptional (Fig 5G) and protein-level (Fig 5I) analyses showed elevated RARRES2 expression in GBM. Immunohistochemistry confirmed a significantly higher proportion of RARRES2 ⁺ cells in the malignant subtype (Fig 5J), consistent with single-cell analysis (S1H Fig). Like RARRES2, the expression of CMKLR1, GPR1, and CCRL2 was higher in GBM than in normal brain or LGG tissues (S9A–C Fig). A meta-analysis of progression-free survival (PFS) using transcriptomic data from TCGA and GEO (datasets with >50 cases) identified RARRES2 as a significant prognostic risk factor (Fig 5H).

Additionally, GSVA and correlation analyses revealed that the activity of glycolipid metabolism–related pathways (such as the PPAR signaling pathway, glycosphingolipid biosynthesis, and glycolysis/gluconeogenesis) was positively correlated with RARRES2 expression (S9D Fig). However, it should be noted that in most glioma cohorts of TCGA and CGGA, RARRES2 expression was significantly negatively correlated with key genes of the fatty acid synthesis pathway (including SREBF2, FASN, ACACA, and ACSL1) but positively correlated with genes involved in long-chain fatty acid synthesis (S9E Fig).

### 3.7. Pan‑cancer analysis of RARRES2 in TCGA cohorts

**Fig 6:**
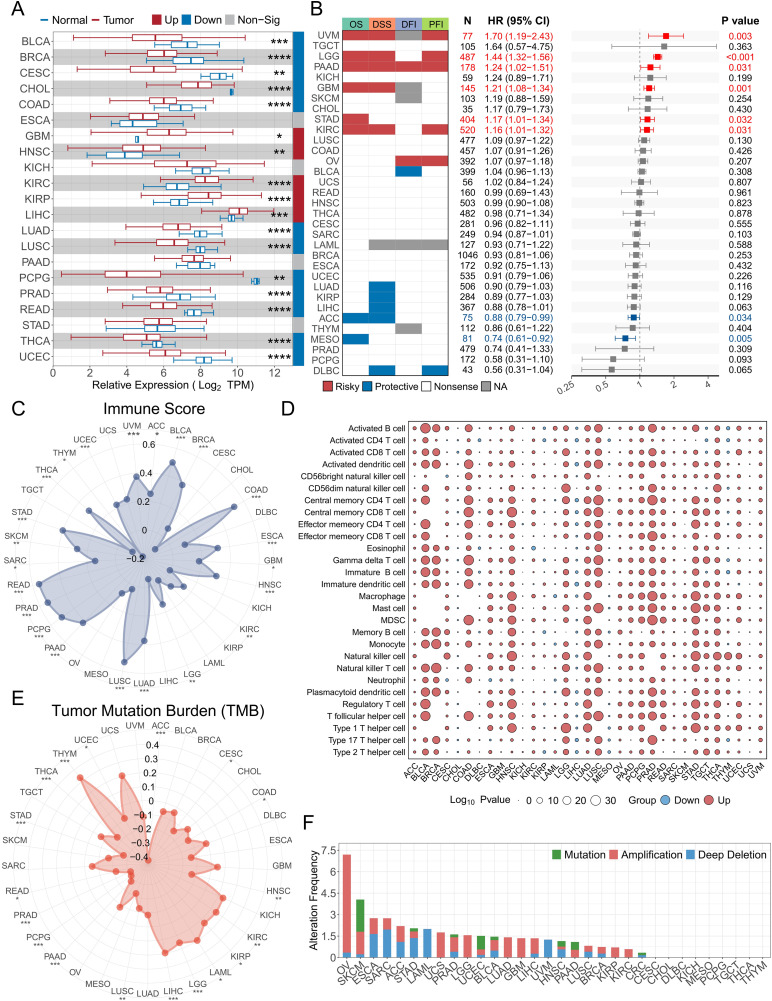
Pan-cancer analysis of RARRES2. (A) RARRES2 expression distribution between tumor and normal tissues across 33 cancer types. (B) Summary of the relationship between RARRES2 expression and OS, DSS, DFI, and PFI based on univariate Cox regression. Red indicates RARRES2 as a risk factor, and blue indicates a protective factor. Only *P* values < 0.05 are shown. (C) Correlation analysis between RARRES2 expression and immune score. (D) Dot plot showing differential infiltration levels between high- and low-RARRES2 expression groups. Circle size represents the P value of the correlation. Red indicates upregulation in the high-expression group, and blue indicates downregulation. (E) Correlation analysis between RARRES2 expression and tumor mutation burden. (F) Analysis of RARRES2 alteration frequency across cancers. (*P* < 0.05, *P < 0.01, **P < 0.001, ***P < 0.0001).

Finally, given that GTEx and HPA data show relatively low RARRES2 expression in normal brain tissue compared to other tissues (S10 Fig), we assessed its dysregulation and prognostic impact across 33 cancer types. Compared to normal tissues, RARRES2 expression was lower in 12 tumor types, including bladder urothelial carcinoma (BLCA), breast invasive carcinoma (BRCA), cervical and endocervical cancer (CESC), cholangiocarcinoma (CHOL), colon adenocarcinoma (COAD), lung adenocarcinoma (LUAD), lung squamous cell carcinoma (LUSC), pheochromocytoma and paraganglioma (PCPG), prostate adenocarcinoma (PRAD), rectum adenocarcinoma (READ), thyroid carcinoma (THCA), and uterine corpus endometrial carcinoma (UCEC), but higher in five tumor tissue types: glioblastoma multiforme (GBM), head and neck squamous cell carcinoma (HNSC), kidney renal clear cell carcinoma (KIRC), kidney papillary cell carcinoma (KIRP), and liver hepatocellular carcinoma (LIHC) (Fig 6A). Univariate Cox regression indicated that RARRES2 could serve as a prognostic risk factor in six cancers, including uveal melanoma (UVM), low-grade glioma (LGG), pancreatic adenocarcinoma (PAAD), glioblastoma multiforme (GBM), stomach adenocarcinoma (STAD), and kidney renal clear cell carcinoma (KIRC) (Fig 6B). Across the TCGA pan-cancer cohort, RARRES2 expression was significantly correlated with the immune score in various cancer types (Fig 6C). Notably, RARRES2 showed both immunostimulatory and immunoinhibitory associations: in cancers like BLCA, BRCA, HNSC, LGG, LUAD, LUSC, PCPG, PRAD, and THCA, RARRES2 was positively correlated with most immunostimulators (e.g., CD40LG, CCL12, TNFSF14), while also positively correlated with certain immunoinhibitors (e.g., CSF1R, IL10, TGFBR1) in BLCA, BRCA, COAD, HNSC, LUAD, LUSC, PRAD, and THCA (S11A–C Fig). Furthermore, RARRES2 showed concurrent positive correlations with both anti-tumor (e.g., activated T cells, NK cells, type 1 helper T cells) and pro-tumor/immunosuppressive (e.g., Tregs, Th2 cells, MDSCs) immune cell infiltrates in most cancer types (S11D Fig). These coordinated patterns suggest a RARRES2-mediated immunoregulatory feedback loop, potentially explaining the paradoxical coexistence of immune activation and suppression in the tumor microenvironment.

Additionally, RARRES2 was significantly correlated with tumor mutation burden (TMB) in ACC, LGG, LIHC, PAAD, PCPG, PRAD, STAD, THCA, and THYM (Fig 6E). Genomic alteration analysis revealed that RARRES2 alterations are relatively uncommon across cancers, with ovarian serous cystadenocarcinoma showing the highest alteration frequency (mostly amplifications) (Fig 6F).

## 4. Discussion

The high heterogeneity of glioma poses significant challenges for classification and treatment strategies. Advances in genetic and biochemical research have revolutionized therapeutic approaches and identified several novel prognostic signatures that may guide treatment decisions [5,38]. In recent years, single-cell RNA sequencing (scRNA-seq) has provided unprecedented insights into tumor biology, enabling the identification and characterization of distinct tumor cell populations and their functional roles. The integration of scRNA-seq with bulk RNA-seq offers the potential for a more comprehensive and precise understanding of glioma pathogenesis and for identifying potential therapeutic targets [36,41].

In our study, we identified 2,078 genes that were highly expressed in three malignant tumor cell clusters; of these, 689 genes were also upregulated in GBM tissue based on bulk RNA-seq. Through univariate Cox regression and LASSO analysis, we pinpointed IGFBP2, MDK, and RARRES2 as key prognostic markers in the TCGA-GBM cohort, a finding validated across five cohorts from the TCGA and CGGA databases. Multivariable Cox regression analyses indicate that our malignant prognostic signature (MPS) retains independent prognostic value after adjustment for IDH mutation status, 1p/19q codeletion, and MGMT promoter methylation.

Insulin-like growth factor (IGF) binding protein 2 (IGFBP2) has been found to be highly expressed in many cancers and participates in tumorigenesis through multiple potential pathways, such as integrin β1/ERK, integrin/ILK/NF-κB, and EGFR/STAT3, among others [42,43]. MDK (midkine) is a neurotrophic growth factor prominently expressed during embryogenesis, and it has emerged as a key player in cancer progression and drug resistance [44]. In GBM patients, upregulated MDK diminishes the survival benefits of temozolomide [45,46], and the MDK/ALK axis regulates the self-renewal of glioma-initiating cells by controlling the autophagic degradation of the transcription factor SOX9 [47]. RARRES2, also known as TIG2 (tubone-induced gene 2) or chemerin, serves as the ligand for CMKLR1 (G protein-coupled receptor chemokine-like receptor 1), GPR1 (G protein-coupled receptor-1), and CCRL2 (chemokine receptor-like 2), which play important roles in adipogenesis, angiogenesis, skin function, and metabolic activity [40]. In hepatocellular carcinoma, chemerin suppresses metastasis through the CMKLR1-PTEN-Akt axis [48]. In glioma, RARRES2 enhances the mesenchymal features of glioblastoma in a CMKLR1-dependent manner [49]. Interestingly, in HepG2 cells, chemerin accelerates hepatocyte lipid accumulation through GPR1 rather than CMKLR1 [50].

We further verified the dysregulation and prognostic efficacy of each MPS gene in five glioma cohorts from TCGA and CGGA. In all cohorts, RARRES2 was a significant prognostic risk factor with an average AUC > 0.7. External cohorts from GEO and our in-house samples also confirmed that RARRES2 is upregulated in GBM compared to LGG and normal tissues, which may be related to altered methylation at the RARRES2 promoter [51].

In melanoma, chemerin-expressing tumors show enhanced infiltration by NK and T cells, with increased ratios of NK/T cells to myeloid-derived suppressor cells or plasmacytoid dendritic cells [40]. Similarly, in four independent glioma cohorts (TCGA and CGGA), we observed that multiple immune- and cancer-related pathways were significantly enriched in high-RARRES2 samples. RARRES2 expression was significantly positively correlated with the abundance of macrophages, NK cells, and CD8 ⁺ T cells. Among these, M2-like macrophages (but not M1-like macrophages) are more closely associated with RARRES2 and are proportionally upregulated and are the main contributor to changes in macrophage infiltration. This suggests that RARRES2 may be linked to the formation of an immunosuppressive microenvironment in glioma. Given the specific distribution of RARRES2 receptors CMKLR1, GPR1, and CCRL2 in myeloid versus malignant cells, we hypothesize that RARRES2 may recruit and polarize macrophages via CMKLR1/CCRL2-mediated paracrine signaling, with a bias toward M2 macrophages, thereby contributing to immunosuppression. However, experimental evidence is needed to confirm this hypothesis. This dual role of RARRES2 in macrophage biology may be a key mechanism underlying its association with glioma progression.

Additionally, notably, in triple-negative breast cancer cells, reduced expression of RARRES2 increases glycerophospholipid levels and decreases levels of triacylglycerols (TAGs) by regulating the PTEN-mTOR-SREBP1 signaling pathway, thereby facilitating the colonization of breast cancer cells in adapting to the brain microenvironment [52]. Zhu et al. also observed passive TAG accumulation after RARRES2 overexpression in hepatic lipid metabolism [50]. In the glioma cohorts from the TCGA and CGGA databases, we observed that the expression level of RARRES2 was significantly correlated with PPAR pathway activity and multiple key genes for fatty acid synthesis. Considering the well-established tight connection between chemerin expression and lipid metabolism [53] and the exaggerated lipogenesis and lipid metabolism found in gliomas [54], we hypothesize that RARRES2 may influence glioma progression through lipid metabolic pathways, a possibility that warrants further functional investigation.

Previous studies have shown that chemerin plays context-dependent roles in cancer biology, acting as both a pro-inflammatory and anti-inflammatory mediator [40]. To explore RARRES2’s clinical and immunological relevance broadly, we analyzed TCGA pan-cancer datasets. RARRES2 was downregulated in tumor tissues of over one-third of cancer types, and elevated RARRES2 levels were significantly associated with poor prognosis in six cancers (UVM, LGG, PAAD, GBM, STAD, KIRC). Intriguingly, high RARRES2 expression correlated with concurrent upregulation of both inflammatory cytokines (e.g., IL-1β, TNF-α) and immunosuppressive markers (e.g., PD-L1, TGF-β) in most cancers. This paradox may reflect heterogeneity in immune microenvironments across tumor types. Gliomas typically have an immune landscape dominated by infiltrating macrophages/microglia [55,56], with fewer lymphoid cells, whereas non-glial tumors have more diverse myeloid and lymphoid populations [55,57–60], many of which express chemerin receptors (e.g., CMKLR1, GPR1) [61,62]. Such microenvironmental diversity likely enables RARRES2 to mediate context-specific immune effects, potentially reconciling its dual association with immune activation and suppression in cancer.

In conclusion, through comprehensive analysis of bulk and single-cell RNA-seq data using various bioinformatics and machine-learning approaches, we developed a robust prognostic signature for glioma. We also examined the dysregulation and functional significance of RARRES2, providing valuable insights for prognostic evaluation and personalized treatment strategies in glioma. However, our study has several limitations. First, reliance on publicly available clinical data may introduce inherent biases. Second, the small size of our in-house validation cohort limits statistical power and calls for validation in larger cohorts. Third, in the drug sensitivity analysis, possibly due to sample heterogeneity or limited sample size, we did not observe a significant negative correlation between any drug’s IC₅₀ and RARRES2 expression across all cohorts. Fourth, although our mouse experiments confirmed RARRES2 expression in glioma tissues, they did not demonstrate a direct role in macrophage polarization or tumor progression; thus, the proposed mechanisms in immune modulation and lipid metabolism remain speculative. Multicenter studies with larger samples and longer follow-up, as well as additional functional experiments are needed to validate our findings and assess their clinical applicability.

## Supporting information

S1 Fig (A) Heatmap displaying the mean expression levels of differentially expressed genes across clusters. (B-C) Boxplots comparing the G2M phase score (B) and S phase score (C) across clusters. (D) Relative ratio of different cell cycle phases in each cluster. (E) Heatmap displaying hallmark term scores in malignant cells and oligodendrocytes. (F-H) Expression distribution of IGFBP2, MDK, and RARRES2 across clusters.
(PNG)

S2 Fig Calibration plots of the malignant score for predicting OS in the TCGA-GBM, TCGA-LGG, mRNAarray 301, mRNAseq 325, and mRNAseq 639 cohorts. (PNG)

S3 Fig Decision curve analysis (DCA) of signature genes and WHO grade for predicting 3-year overall survival in the mRNAarray 301 (A-C), mRNAseq 325 (D-F), and mRNAseq 639 (G-I) cohorts. (PNG)

S4 Fig Relationship between IC50 values and signature gene expression. Dot plots showing the relationship between IC50 values and IGFBP2 (A), MDK (B), and RARRES2 (C) in the TCGA-GBM, TCGA-LGG, mRNAseq 325, and mRNAseq 639 cohorts. Circle size represents the P value of the correlation. Golden yellow indicates a positive correlation, and dark green indicates a negative correlation.
(PNG)

S5 Fig Correlation analysis of RARRES2, MDK, and IGFBP2 expression levels with immune scores in the TCGA-GBM, TCGA-LGG, mRNAseq 325, and mRNAseq 639 cohorts. (PNG)

S6 Fig Correlation between signature gene expression and immune cell infiltration. Dot plots representing the correlation between the expression of three signature genes and immune cell infiltration abundance. Circle size represents the P value, and color indicates the correlation coefficient.
(PNG)

S7 Fig Cross-algorithm consistency of immune cell infiltration estimates in TCGA-LGG and TCGA-GBM cohorts. Spearman correlation coefficients among six deconvolution algorithms (CIBERSORT, TIMER, xCell, MCP-counter, EPIC, and quanTIseq) for NK cells and CD8 ⁺ T cells in (A) TCGA-LGG and (C) TCGA-GBM, and for monocytes and macrophages in (B) TCGA-LGG and (D) TCGA-GBM. Color intensity represents correlation strength, with red indicating positive correlation. Monocytes and macrophages show strong cross-algorithm concordance, supporting the robustness of their association with RARRES2 expression.
(PNG)

S8 Fig GSEA analysis of immune cell gene signatures between high- and low-RARRES2 groups. (A) Venn diagram of significant immune-related gene signatures in the TCGA-GBM, TCGA-LGG, mRNAseq 325, and mRNAseq 639 cohorts. (B) NES bar plot of GIM (glioma-infiltrating microglia/macrophage), M1 macrophages, and M2 macrophages. (C-F) Distribution of immune cell subset infiltration between high- and low-RARRES2 groups. (G-J) Importance of immune cells for RARRES2 expression.
(PNG)

S9 Fig Analysis of RARRES2 receptor expression and pathway activities. (A-C) Expression distribution of CMKLR1 (A), GPR1 (B), and CCRL2 (C) in normal brain and glioma tissues in three independent GEO cohorts. (D) Correlation analysis between RARRES2 expression levels and KEGG pathway activities in the TCGA-GBM, TCGA-LGG, mRNAseq 325, and mRNAseq 639 cohorts. (E) Correlation analysis between RARRES2 expression levels and fatty acid metabolism genes in the same cohorts.
(PNG)

S10 Fig (A-B) RARRES2 expression distribution in normal tissues from the GTEx (A) and HPA (B) databases. (C) RARRES2 expression distribution across normal cell types in the HPA database.
(PNG)

S11 Fig Correlation between RARRES2 and immune regulators. Dot plots depicting associations of RARRES2 with (A) immunoinhibitors, (B) immunostimulators, and (C) immune checkpoint genes. Circle size represents the P value, and color denotes the Spearman correlation coefficient. (D) Correlation heatmap showing associations of RARRES2 with infiltration of anti-tumor immune cells (ActCD4, ActCD8, TcmCD4, TcmCD8, TemCD4, TemCD8, Th1, Th17, ActDC, CD56briNK, NK, NKT) and pro-tumor immunosuppressive cells (Treg, Th2, CD56dimNK, imDC, TAM, MDSC, neutrophils, and pDC).
(PNG)

S1 Table scRNA-seq cell type marker genes. (XLSX)

S2 Table Differentially expressed genes in each scRNA-seq cluster. (XLSX)

S3 Table Multivariable Cox proportional hazards analysis of MPS and clinical/molecular covariates across five glioma cohorts. (XLSX)

S4 Table Multivariable Cox regression analysis of MPS adjusted for molecular covariates across five glioma cohorts. (XLSX)

## References

[1] Ostrom QT, Bauchet L, Davis FG, Deltour I, Fisher JL, Langer CE, et al. The epidemiology of glioma in adults: a “state of the science” review. Neuro Oncol. 2014;16(7):896–913. doi: 10.1093/neuonc/nou087 24842956 PMC4057143

[2] Gritsch S, Batchelor TT, Gonzalez Castro LN. Diagnostic, therapeutic, and prognostic implications of the 2021 World Health Organization classification of tumors of the central nervous system. Cancer. 2022;128(1):47–58. doi: 10.1002/cncr.33918 34633681

[3] Wesseling P, Capper D. WHO 2016 Classification of gliomas. Neuropathol Appl Neurobiol. 2018;44(2):139–50. doi: 10.1111/nan.12432 28815663

[4] Li N, Hao S, Cao X, Lin Y, Li Y, Dai T, et al. Significance of radiation therapy in frontal glioblastoma patients and exploration of optimal treatment modality: a real-world multiple-center study based on propensity score matching. Quant Imaging Med Surg. 2024;14(10):7576–86. doi: 10.21037/qims-23-1871 39429582 PMC11485375

[5] Śledzińska P, Bebyn MG, Furtak J, Kowalewski J, Lewandowska MA. Prognostic and Predictive Biomarkers in Gliomas. Int J Mol Sci. 2021;22(19):10373. doi: 10.3390/ijms221910373 34638714 PMC8508830

[6] Kricha A, Bouchmaa N, Ben Mkaddem S, Abbaoui A, Ben Mrid R, El Fatimy R. Glioblastoma-associated macrophages: A key target in overcoming glioblastoma therapeutic resistance. Cytokine Growth Factor Rev. 2024;80:97–108. doi: 10.1016/j.cytogfr.2024.10.009 39510901

[7] Sanai N, Chang S, Berger MS. Low-grade gliomas in adults. J Neurosurg. 2011;115(5):948–65. doi: 10.3171/2011.7.JNS101238 22043865

[8] Shaw EG, Berkey B, Coons SW, Bullard D, Brachman D, Buckner JC, et al. Recurrence following neurosurgeon-determined gross-total resection of adult supratentorial low-grade glioma: results of a prospective clinical trial. J Neurosurg. 2008;109(5):835–41. doi: 10.3171/JNS/2008/109/11/0835 18976072 PMC3833272

[9] Murphy ES, Leyrer CM, Parsons M, Suh JH, Chao ST, Yu JS, et al. Risk Factors for Malignant Transformation of Low-Grade Glioma. Int J Radiat Oncol Biol Phys. 2018;100(4):965–71. doi: 10.1016/j.ijrobp.2017.12.258 29485076

[10] Delgado-López PD, Corrales-García EM, Martino J, Lastra-Aras E, Dueñas-Polo MT. Diffuse low-grade glioma: a review on the new molecular classification, natural history and current management strategies. Clin Transl Oncol. 2017;19(8):931–44. doi: 10.1007/s12094-017-1631-4 28255650

[11] Jansen E, Hamisch C, Ruess D, Heiland DH, Goldbrunner R, Ruge MI, et al. Observation after surgery for low grade glioma: long-term outcome in the light of the 2016 WHO classification. J Neurooncol. 2019;145(3):501–7. doi: 10.1007/s11060-019-03316-7 31621043

[12] Witthayanuwat S, Pesee M, Supaadirek C, Supakalin N, Thamronganantasakul K, Krusun S. Survival Analysis of Glioblastoma Multiforme. Asian Pacific Journal of Cancer Prevention. 2018;19(9):2613–7. doi: 10.22034/APJCP.2018.19.9.2613 30256068 PMC6249474

[13] Wach J, Vychopen M, Kuhnapfel A, Seidel C, Guresir E. A systematic review and meta-analysis of supramarginal resection versus gross total resection in glioblastoma: can we enhance progression-free survival time and preserve postoperative safety? Cancers. 2023;15(6). doi: 10.3390/cancers15061772 36980659 PMC10046815

[14] Poursaeed R, Mohammadzadeh M, Safaei AA. Survival prediction of glioblastoma patients using machine learning and deep learning: a systematic review. BMC Cancer. 2024;24(1):1581. doi: 10.1186/s12885-024-13320-4 39731064 PMC11674357

[15] Kalluri AL, Lee JH, Lucas C-HG, Rincon-Torroella J, Bettegowda C. Implications of molecular classifications in glioma surgery. J Neurooncol. 2025;171(3):559–69. doi: 10.1007/s11060-024-04883-0 39532825

[16] Sahraeian SME, Mohiyuddin M, Sebra R, Tilgner H, Afshar PT, Au KF, et al. Gaining comprehensive biological insight into the transcriptome by performing a broad-spectrum RNA-seq analysis. Nat Commun. 2017;8(1):59. doi: 10.1038/s41467-017-00050-4 28680106 PMC5498581

[17] Ziegenhain C, Vieth B, Parekh S, Reinius B, Guillaumet-Adkins A, Smets M, et al. Comparative Analysis of Single-Cell RNA Sequencing Methods. Mol Cell. 2017;65(4):631-643.e4. doi: 10.1016/j.molcel.2017.01.023 28212749

[18] Marczyk M, Fu C, Lau R, Du L, Trevarton AJ, Sinn BV, et al. The impact of RNA extraction method on accurate RNA sequencing from formalin-fixed paraffin-embedded tissues. BMC Cancer. 2019;19(1):1189. doi: 10.1186/s12885-019-6363-0 31805884 PMC6896723

[19] Kwong LN, De Macedo MP, Haydu L, Joon AY, Tetzlaff MT, Calderone TL, et al. Biological Validation of RNA Sequencing Data from Formalin-Fixed Paraffin-Embedded Primary Melanomas. JCO Precis Oncol. 2018;2018:10.1200/PO.17.00259. doi: 10.1200/PO.17.00259 31058252 PMC6498859

[20] Hao Y, Hao S, Andersen-Nissen E, Mauck WM 3rd, Zheng S, Butler A, et al. Integrated analysis of multimodal single-cell data. Cell. 2021;184(13):3573-3587.e29. doi: 10.1016/j.cell.2021.04.048 34062119 PMC8238499

[21] Korsunsky I, Millard N, Fan J, Slowikowski K, Zhang F, Wei K, et al. Fast, sensitive and accurate integration of single-cell data with Harmony. Nat Methods. 2019;16(12):1289–96. doi: 10.1038/s41592-019-0619-0 31740819 PMC6884693

[22] Abdelfattah N, Kumar P, Wang C, Leu J-S, Flynn WF, Gao R, et al. Single-cell analysis of human glioma and immune cells identifies S100A4 as an immunotherapy target. Nat Commun. 2022;13(1):767. doi: 10.1038/s41467-022-28372-y 35140215 PMC8828877

[23] Liang Y, Zhang R, Biswas S, Bu Q, Xu Z, Qiao L, et al. Integrated single-cell transcriptomics reveals the hypoxia-induced inflammation-cancer transformation in NASH-derived hepatocellular carcinoma. Cell Prolif. 2024;57(4):e13576. doi: 10.1111/cpr.13576 37994257 PMC10984103

[24] Patel AP, Tirosh I, Trombetta JJ, Shalek AK, Gillespie SM, Wakimoto H, et al. Single-cell RNA-seq highlights intratumoral heterogeneity in primary glioblastoma. Science. 2014;344(6190):1396–401. doi: 10.1126/science.1254257 24925914 PMC4123637

[25] Xiao Y, Wang Z, Zhao M, Deng Y, Yang M, Su G, et al. Single-Cell Transcriptomics Revealed Subtype-Specific Tumor Immune Microenvironments in Human Glioblastomas. Front Immunol. 2022;13:914236. doi: 10.3389/fimmu.2022.914236 35669791 PMC9163377

[26] Love MI, Huber W, Anders S. Moderated estimation of fold change and dispersion for RNA-seq data with DESeq2. Genome Biol. 2014;15(12):550. doi: 10.1186/s13059-014-0550-8 25516281 PMC4302049

[27] Engebretsen S, Bohlin J. Statistical predictions with glmnet. Clin Epigenetics. 2019;11(1):123. doi: 10.1186/s13148-019-0730-1 31443682 PMC6708235

[28] Blanche P, Dartigues J-F, Jacqmin-Gadda H. Estimating and comparing time-dependent areas under receiver operating characteristic curves for censored event times with competing risks. Stat Med. 2013;32(30):5381–97. doi: 10.1002/sim.5958 24027076

[29] Liberzon A, Birger C, Thorvaldsdóttir H, Ghandi M, Mesirov JP, Tamayo P. The Molecular Signatures Database (MSigDB) hallmark gene set collection. Cell Syst. 2015;1(6):417–25. doi: 10.1016/j.cels.2015.12.004 26771021 PMC4707969

[30] Wu T, Hu E, Xu S, Chen M, Guo P, Dai Z. clusterProfiler 4.0: A universal enrichment tool for interpreting omics data. Innovation (Camb). 2021;2(3):100141. doi: 10.1016/j.xinn.2021.100141 34557778 PMC8454663

[31] Hänzelmann S, Castelo R, Guinney J. GSVA: gene set variation analysis for microarray and RNA-seq data. BMC Bioinformatics. 2013;14:7. doi: 10.1186/1471-2105-14-7 23323831 PMC3618321

[32] Maeser D, Gruener RF, Huang RS. oncoPredict: an R package for predicting in vivo or cancer patient drug response and biomarkers from cell line screening data. Brief Bioinform. 2021;22(6):bbab260. doi: 10.1093/bib/bbab260 34260682 PMC8574972

[33] Yoshihara K, Shahmoradgoli M, Martínez E, Vegesna R, Kim H, Torres-Garcia W, et al. Inferring tumour purity and stromal and immune cell admixture from expression data. Nat Commun. 2013;4:2612. doi: 10.1038/ncomms3612 24113773 PMC3826632

[34] Zeng D, Fang Y, Qiu W, Luo P, Wang S, Shen R, et al. Enhancing immuno-oncology investigations through multidimensional decoding of tumor microenvironment with IOBR 2.0. Cell Rep Methods. 2024;4(12):100910. doi: 10.1016/j.crmeth.2024.100910 39626665 PMC11704618

[35] Li T, Fu J, Zeng Z, Cohen D, Li J, Chen Q, et al. TIMER2.0 for analysis of tumor-infiltrating immune cells. Nucleic Acids Res. 2020;48(W1):W509–14. doi: 10.1093/nar/gkaa407 32442275 PMC7319575

[36] Yuan J, Levitin HM, Frattini V, Bush EC, Boyett DM, Samanamud J, et al. Single-cell transcriptome analysis of lineage diversity in high-grade glioma. Genome Med. 2018;10(1):57. doi: 10.1186/s13073-018-0567-9 30041684 PMC6058390

[37] Eckel-Passow JE, Lachance DH, Molinaro AM, Walsh KM, Decker PA, Sicotte H. Glioma Groups Based on 1p/19q, IDH, and TERT Promoter Mutations in Tumors. N Engl J Med. 2015;372(26):2499–508. doi: 10.1056/NEJMoa1407279 26061753 PMC4489704

[38] Chen R, Smith-Cohn M, Cohen AL, Colman H. Glioma Subclassifications and Their Clinical Significance. Neurotherapeutics. 2017;14(2):284–97. doi: 10.1007/s13311-017-0519-x 28281173 PMC5398991

[39] Hu Y, Wang Y, Zhi L, Yu L, Hu X, Shen Y, et al. SDC4 protein action and related key genes in nonhealing diabetic foot ulcers based on bioinformatics analysis and machine learning. Int J Biol Macromol. 2024;283(Pt 2):137789. doi: 10.1016/j.ijbiomac.2024.137789 39557273

[40] Shin WJ, Zabel BA, Pachynski RK. Mechanisms and Functions of Chemerin in Cancer: Potential Roles in Therapeutic Intervention. Front Immunol. 2018;9:2772. doi: 10.3389/fimmu.2018.02772 30555465 PMC6283908

[41] Fan J, Slowikowski K, Zhang F. Single-cell transcriptomics in cancer: computational challenges and opportunities. Exp Mol Med. 2020;52(9):1452–65. doi: 10.1038/s12276-020-0422-0 32929226 PMC8080633

[42] Zhang B, Hong C-Q, Luo Y-H, Wei L-F, Luo Y, Peng Y-H, et al. Prognostic value of IGFBP2 in various cancers: a systematic review and meta-analysis. Cancer Med. 2022;11(16):3035–47. doi: 10.1002/cam4.4680 35546443 PMC9385590

[43] Li T, Forbes ME, Fuller GN, Li J, Yang X, Zhang W. IGFBP2: integrative hub of developmental and oncogenic signaling network. Oncogene. 2020;39(11):2243–57. doi: 10.1038/s41388-020-1154-2 31925333 PMC7347283

[44] Jiao J, Yin M, Wang Z, Hu B, Chi J, Lu L, et al. An oriented self-assembly biosensor with built-in error-checking for precise midkine detection in cancer diagnosis and prognosis evaluation. Biosens Bioelectron. 2025;268:116905. doi: 10.1016/j.bios.2024.116905 39504885

[45] Zhao Y, Qin J, Yu D, Liu Y, Song D, Tian K, et al. Polymer-locking fusogenic liposomes for glioblastoma-targeted siRNA delivery and CRISPR-Cas gene editing. Nat Nanotechnol. 2024;19(12):1869–79. doi: 10.1038/s41565-024-01769-0 39209994

[46] Yu X, Zhou Z, Tang S, Zhang K, Peng X, Zhou P, et al. MDK induces temozolomide resistance in glioblastoma by promoting cancer stem-like properties. Am J Cancer Res. 2022;12(10):4825–39. 36381313 PMC9641408

[47] López-Valero I, Dávila D, González-Martínez J, Salvador-Tormo N, Lorente M, Saiz-Ladera C, et al. Midkine signaling maintains the self-renewal and tumorigenic capacity of glioma initiating cells. Theranostics. 2020;10(11):5120–36. doi: 10.7150/thno.41450 32308772 PMC7163450

[48] Li J-J, Yin H-K, Guan D-X, Zhao J-S, Feng Y-X, Deng Y-Z, et al. Chemerin suppresses hepatocellular carcinoma metastasis through CMKLR1-PTEN-Akt axis. Br J Cancer. 2018;118(10):1337–48. doi: 10.1038/s41416-018-0077-y 29717200 PMC5959946

[49] Wu J, Shen S, Liu T, Ren X, Zhu C, Liang Q, et al. Chemerin enhances mesenchymal features of glioblastoma by establishing autocrine and paracrine networks in a CMKLR1-dependent manner. Oncogene. 2022;41(21):3024–36. doi: 10.1038/s41388-022-02295-w 35459783 PMC9122825

[50] Zhu L, Huang J, Wang Y, Yang Z, Chen X. Chemerin causes lipid metabolic imbalance and induces passive lipid accumulation in human hepatoma cell line via the receptor GPR1. Life Sci. 2021;278:119530. doi: 10.1016/j.lfs.2021.119530 33887347

[51] Wang H, Wang X, Xu L, Zhang J. RARRES2 is Downregulated in Isocitrate Dehydrogenase 1 Mutant Glioma Patients and Served as an Unfavorable Prognostic Factor of Glioma. World Neurosurg. 2023;176:e610–22. doi: 10.1016/j.wneu.2023.05.109 37271257

[52] Li Y-Q, Sun F-Z, Li C-X, Mo H-N, Zhou Y-T, Lv D, et al. RARRES2 regulates lipid metabolic reprogramming to mediate the development of brain metastasis in triple negative breast cancer. Mil Med Res. 2023;10(1):34. doi: 10.1186/s40779-023-00470-y 37491281 PMC10369725

[53] Goralski KB, McCarthy TC, Hanniman EA, Zabel BA, Butcher EC, Parlee SD, et al. Chemerin, a novel adipokine that regulates adipogenesis and adipocyte metabolism. J Biol Chem. 2007;282(38):28175–88. doi: 10.1074/jbc.M700793200 17635925

[54] Guo D, Bell EH, Chakravarti A. Lipid metabolism emerges as a promising target for malignant glioma therapy. CNS Oncol. 2013;2(3):289–99. doi: 10.2217/cns.13.20 24159371 PMC3804348

[55] Sarantopoulos A, Ene C, Aquilanti E. Therapeutic approaches to modulate the immune microenvironment in gliomas. NPJ Precis Oncol. 2024;8(1):241. doi: 10.1038/s41698-024-00717-4 39443641 PMC11500177

[56] Glass R, Synowitz M. CNS macrophages and peripheral myeloid cells in brain tumours. Acta Neuropathol. 2014;128(3):347–62. doi: 10.1007/s00401-014-1274-2 24722970

[57] Laumont CM, Banville AC, Gilardi M, Hollern DP, Nelson BH. Tumour-infiltrating B cells: immunological mechanisms, clinical impact and therapeutic opportunities. Nat Rev Cancer. 2022;22(7):414–30. doi: 10.1038/s41568-022-00466-1 35393541 PMC9678336

[58] Cheng S, Li Z, Gao R, Xing B, Gao Y, Yang Y, et al. A pan-cancer single-cell transcriptional atlas of tumor infiltrating myeloid cells. Cell. 2021;184(3):792-809.e23. doi: 10.1016/j.cell.2021.01.010 33545035

[59] Tang F, Li J, Qi L, Liu D, Bo Y, Qin S, et al. A pan-cancer single-cell panorama of human natural killer cells. Cell. 2023;186(19):4235-4251.e20. doi: 10.1016/j.cell.2023.07.034 37607536

[60] Zheng L, Qin S, Si W, Wang A, Xing B, Gao R, et al. Pan-cancer single-cell landscape of tumor-infiltrating T cells. Science. 2021;374(6574):abe6474. doi: 10.1126/science.abe6474 34914499

[61] Bondue B, Wittamer V, Parmentier M. Chemerin and its receptors in leukocyte trafficking, inflammation and metabolism. Cytokine Growth Factor Rev. 2011;22(5–6):331–8. doi: 10.1016/j.cytogfr.2011.11.004 22119008

[62] Ballet R, LaJevic M, Huskey-Mullin N, Roach R, Brulois K, Huang Y, et al. Chemerin triggers migration of a CD8 T cell subset with natural killer cell functions. Mol Ther. 2023;31(10):2887–900. doi: 10.1016/j.ymthe.2023.08.015 37641406 PMC10556222

