# Supplementary figures and images for: Characterizing malignant prognostic signatures in primary glioma based on single-cell and bulk transcriptome sequencing
Source: PLoS One. 2026 Jun 5;21(6):e0349749. doi: 10.1371/journal.pone.0349749 (PMC13240926; doi:10.1371/journal.pone.0349749)

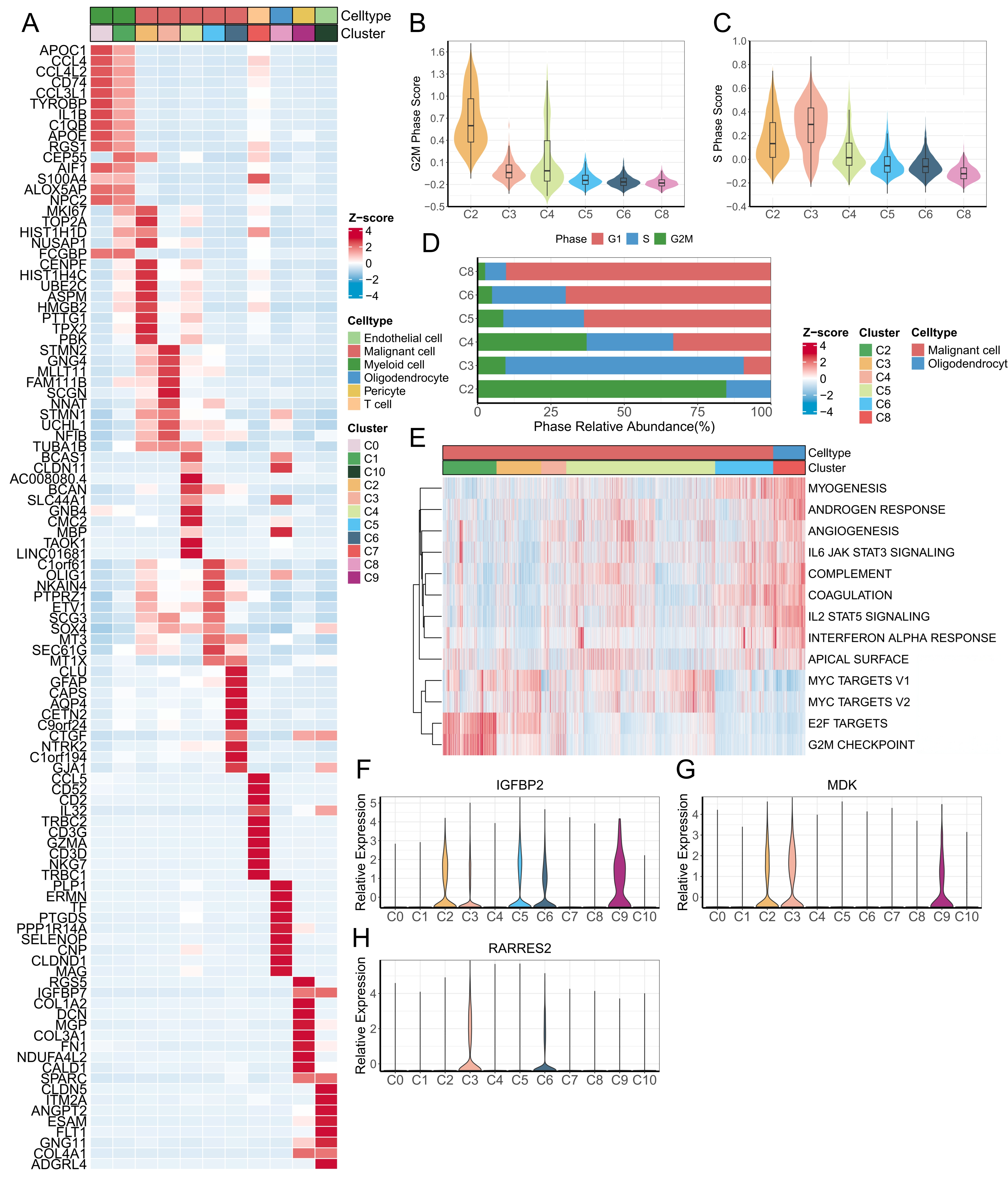

Supplement: S1 Fig — (B-C) Boxplots comparing the G2M phase score (B) and S phase score (C) across clusters. (D) Relative ratio of different cell cycle phases in each cluster. (E) Heatmap displaying hallmark term scores in malignant cells and oligodendrocytes. (F-H) Expression distribution of IGFBP2, MDK, and RARRES2 across clusters. (PNG) [file pone.0349749.s001.png]

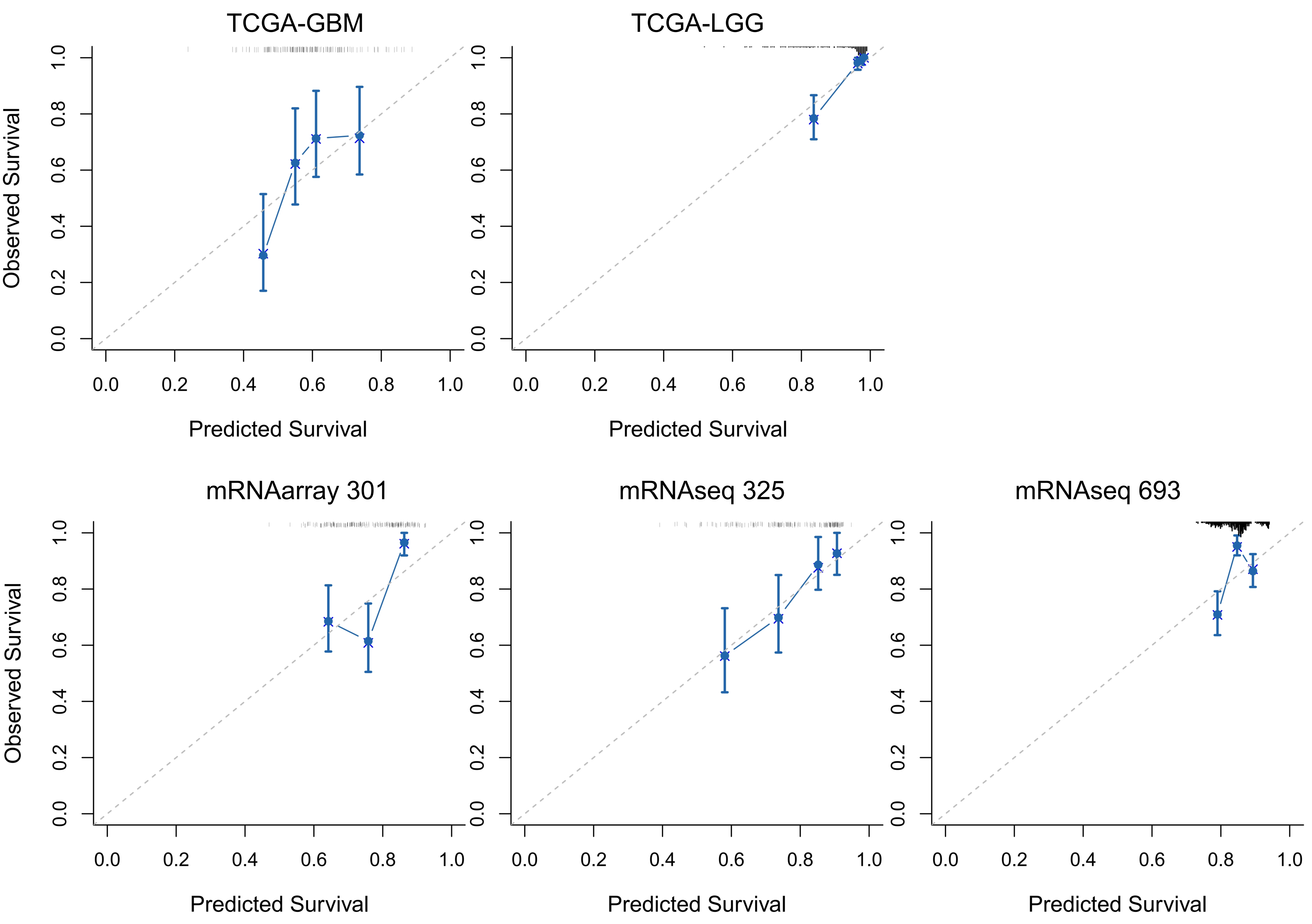

Supplement: S2 Fig — (PNG) [file pone.0349749.s002.png]

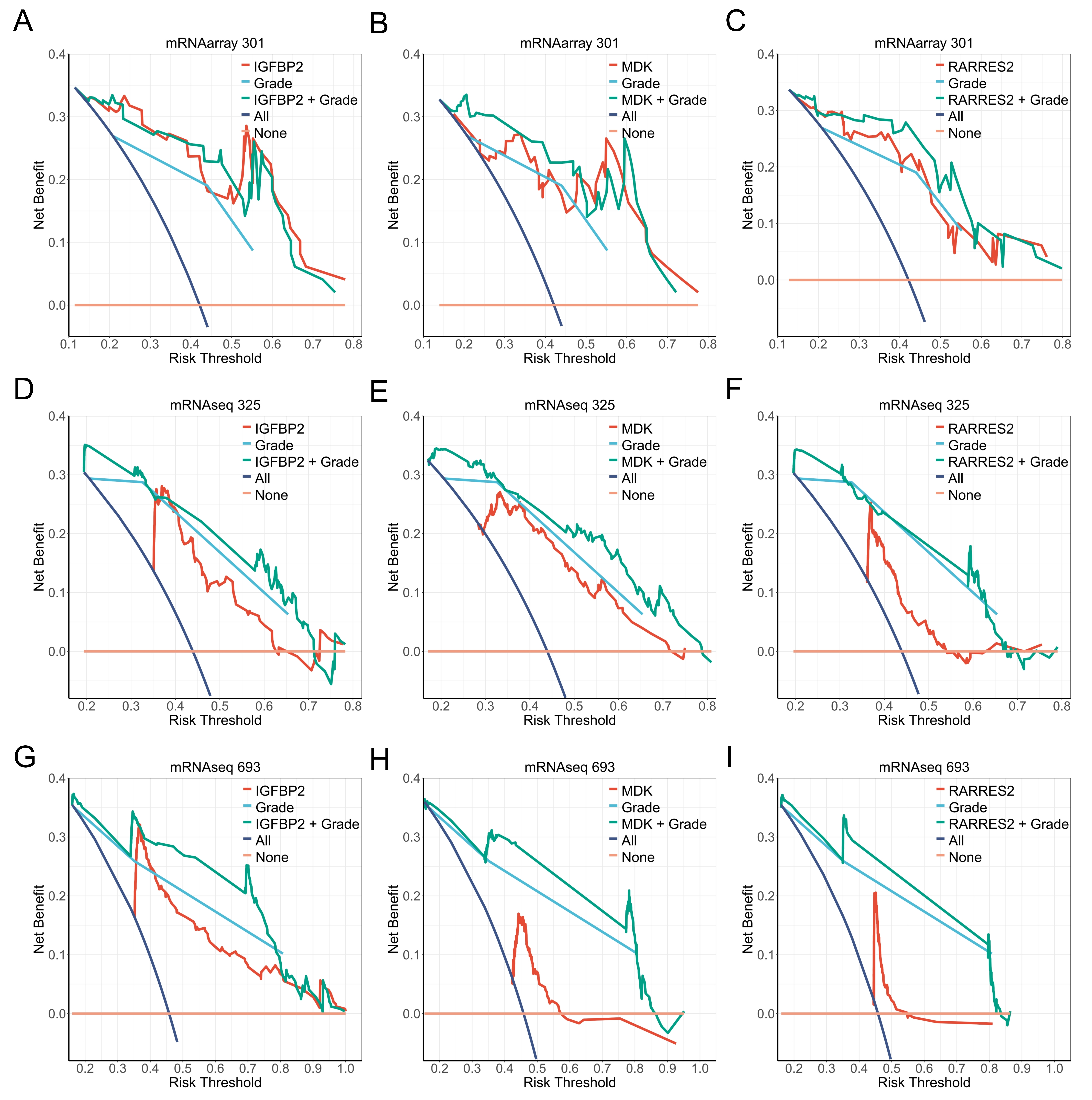

Supplement: S3 Fig — (PNG) [file pone.0349749.s003.png]

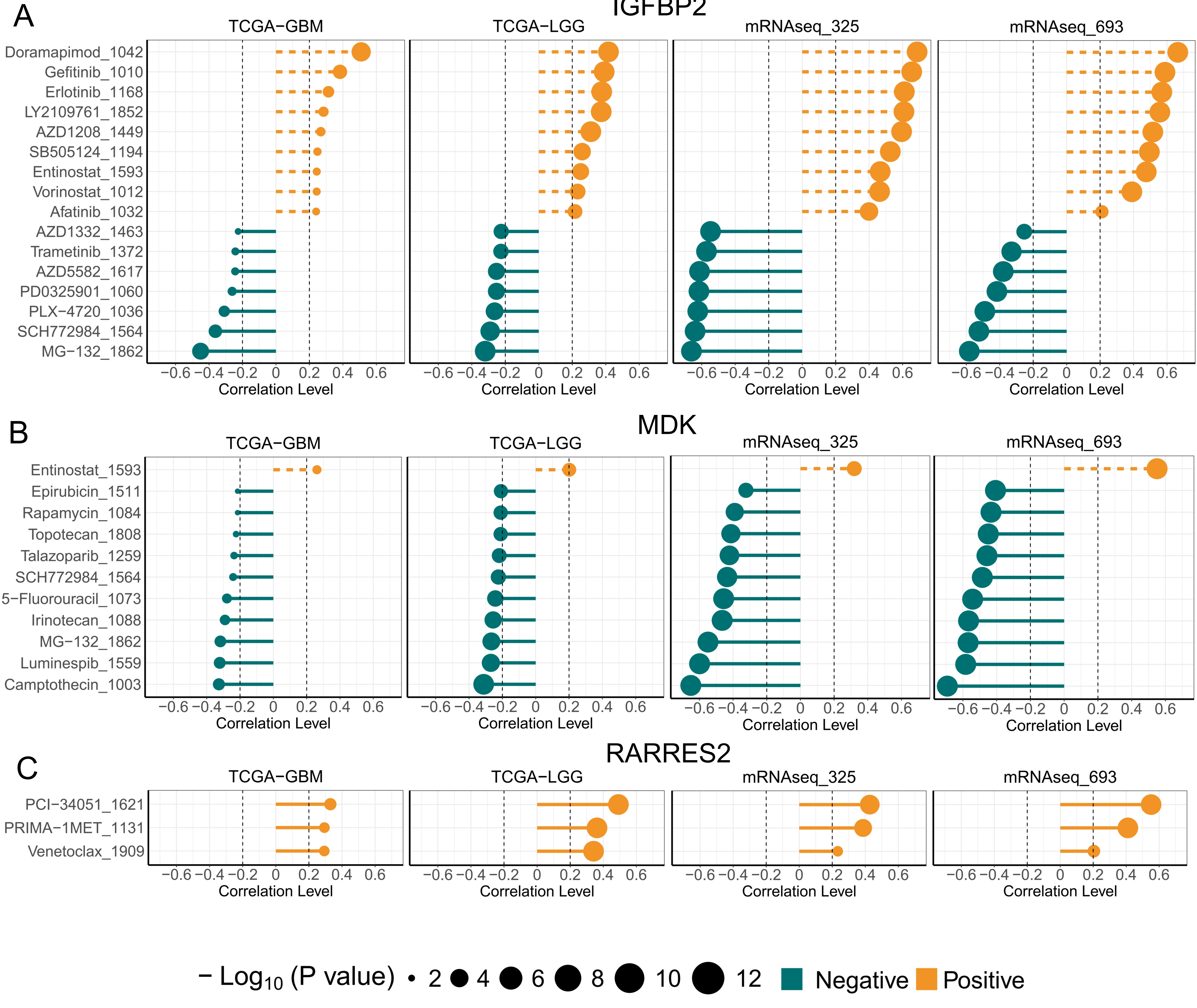

Supplement: S4 Fig — Dot plots showing the relationship between IC50 values and IGFBP2 (A), MDK (B), and RARRES2 (C) in the TCGA-GBM, TCGA-LGG, mRNAseq 325, and mRNAseq 639 cohorts. Circle size represents the P value of the correlation. Golden yellow indicates a positive correlation, and dark green indicates a negative correlation. (PNG) [file pone.0349749.s004.png]

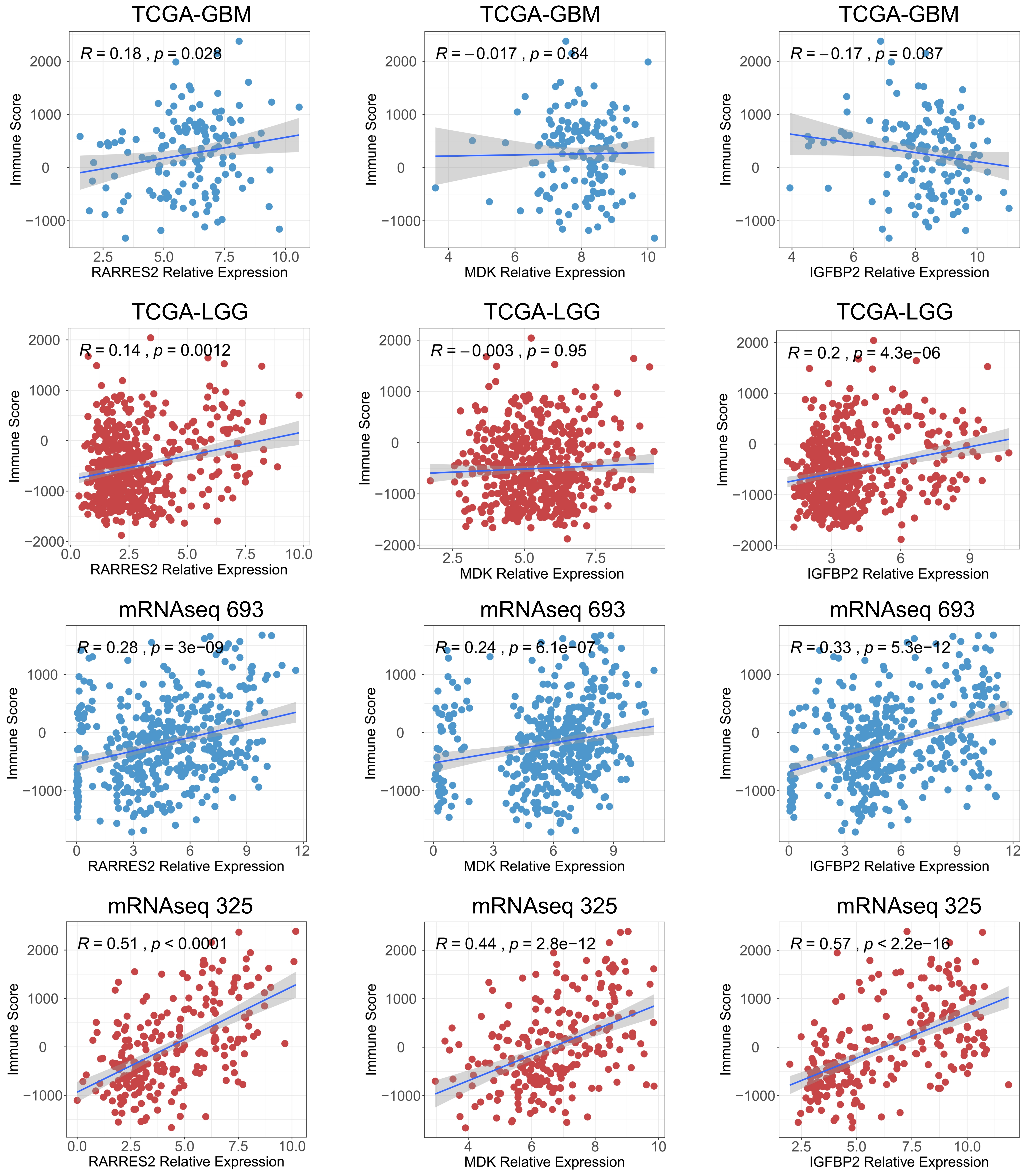

Supplement: S5 Fig — (PNG) [file pone.0349749.s005.png]

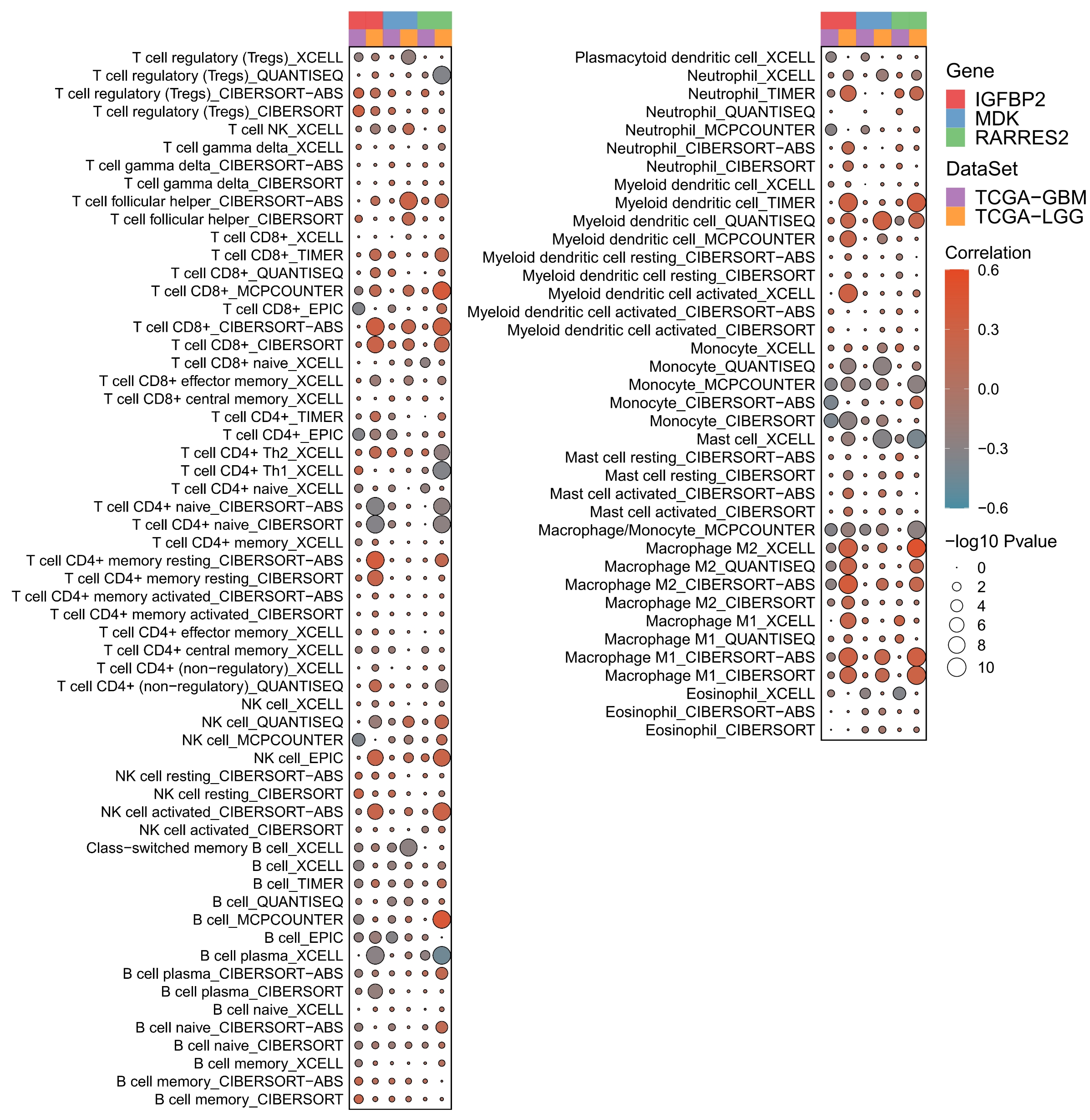

Supplement: S6 Fig — Dot plots representing the correlation between the expression of three signature genes and immune cell infiltration abundance. Circle size represents the P value, and color indicates the correlation coefficient. (PNG) [file pone.0349749.s006.png]

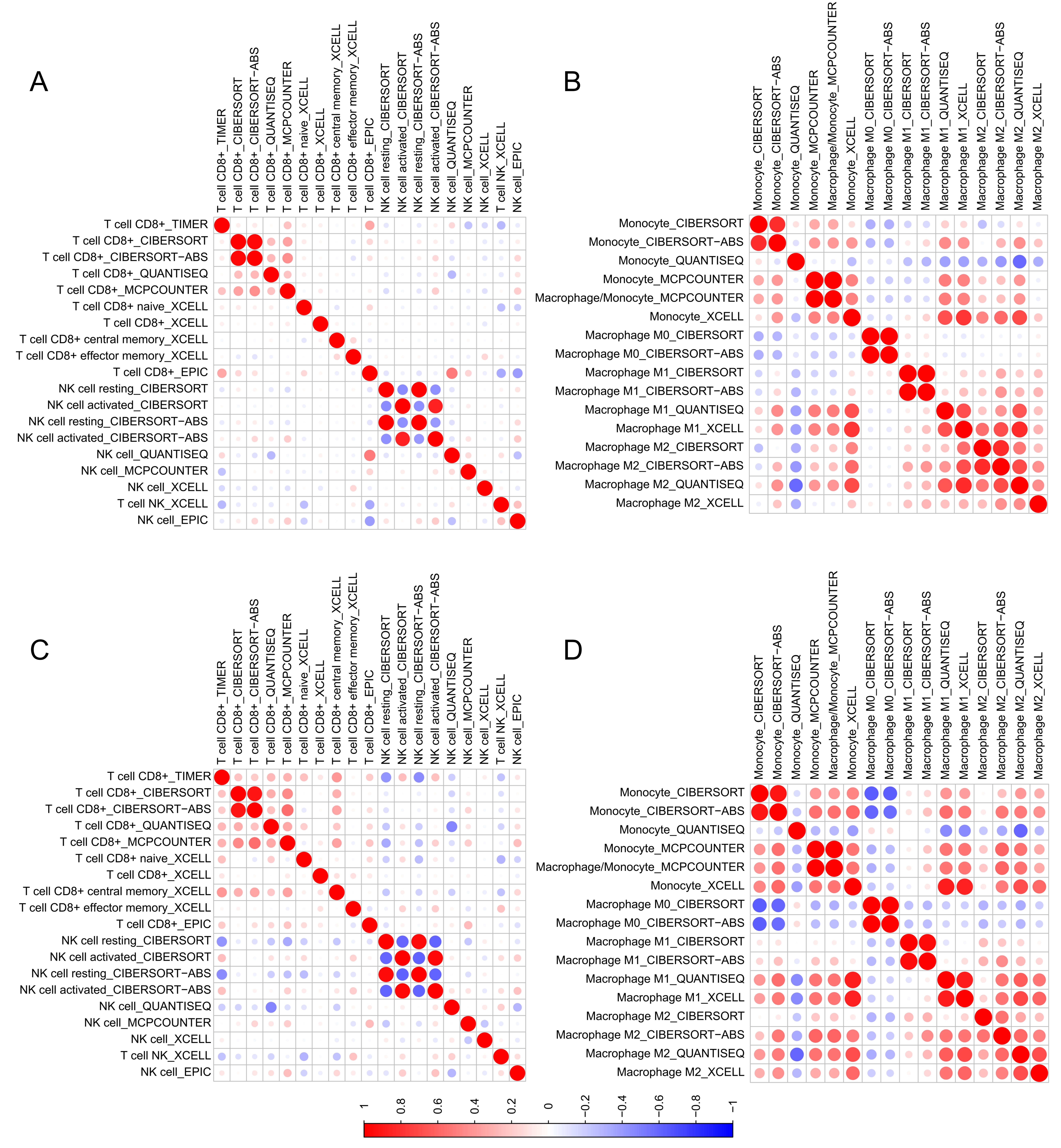

Supplement: S7 Fig — Spearman correlation coefficients among six deconvolution algorithms (CIBERSORT, TIMER, xCell, MCP-counter, EPIC, and quanTIseq) for NK cells and CD8 ⁺ T cells in (A) TCGA-LGG and (C) TCGA-GBM, and for monocytes and macrophages in (B) TCGA-LGG and (D) TCGA-GBM. Color intensity represents correlation strength, with red indicating positive correlation. Monocytes and macrophages show strong cross-algorithm concordance, supporting the robustness of their association with RARRES2 expression. (PNG) [file pone.0349749.s007.png]

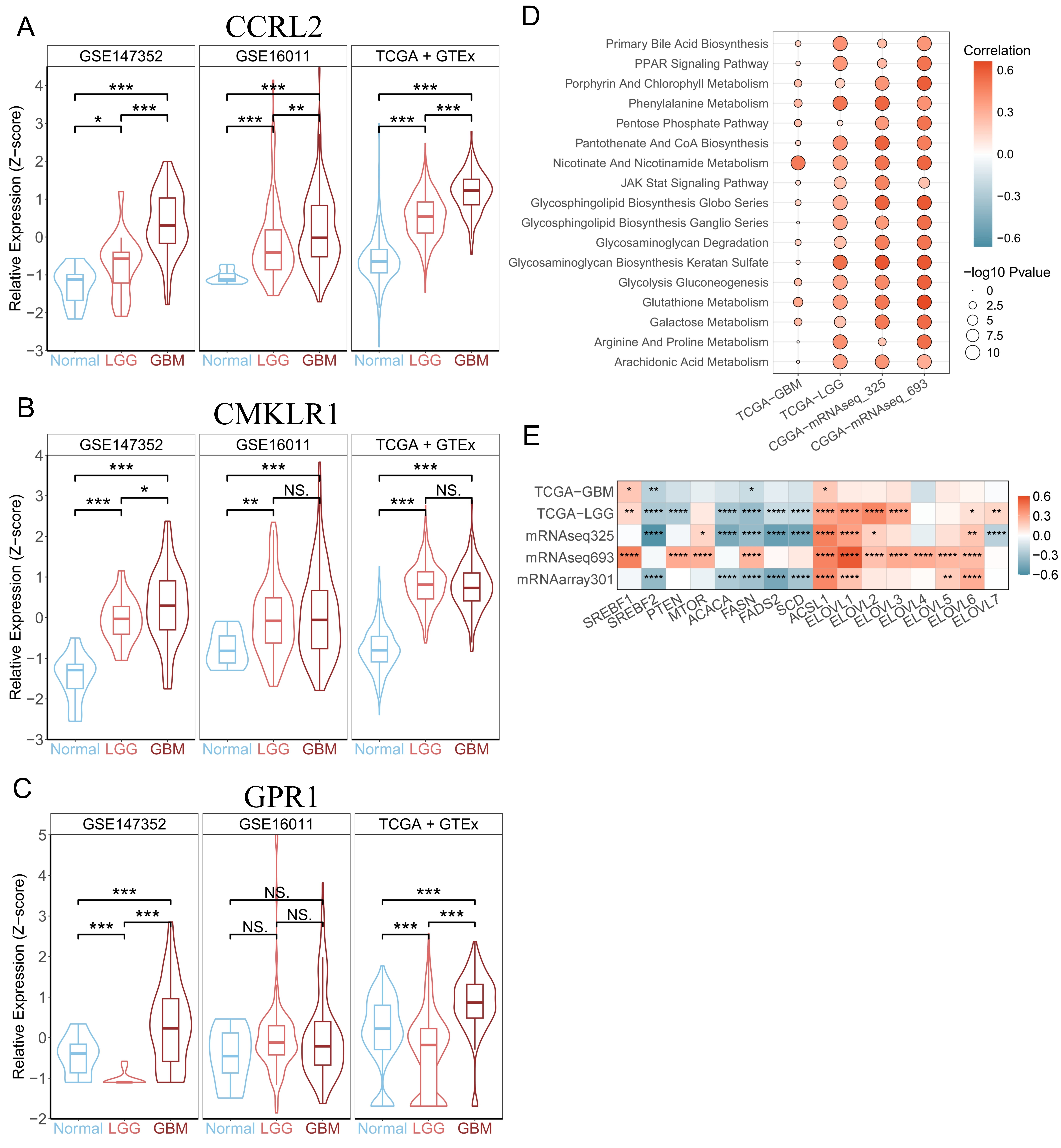

Supplement: S9 Fig — (A-C) Expression distribution of CMKLR1 (A), GPR1 (B), and CCRL2 (C) in normal brain and glioma tissues in three independent GEO cohorts. (D) Correlation analysis between RARRES2 expression levels and KEGG pathway activities in the TCGA-GBM, TCGA-LGG, mRNAseq 325, and mRNAseq 639 cohorts. (E) Correlation analysis between RARRES2 expression levels and fatty acid metabolism genes in the same cohorts. (PNG) [file pone.0349749.s009.png]

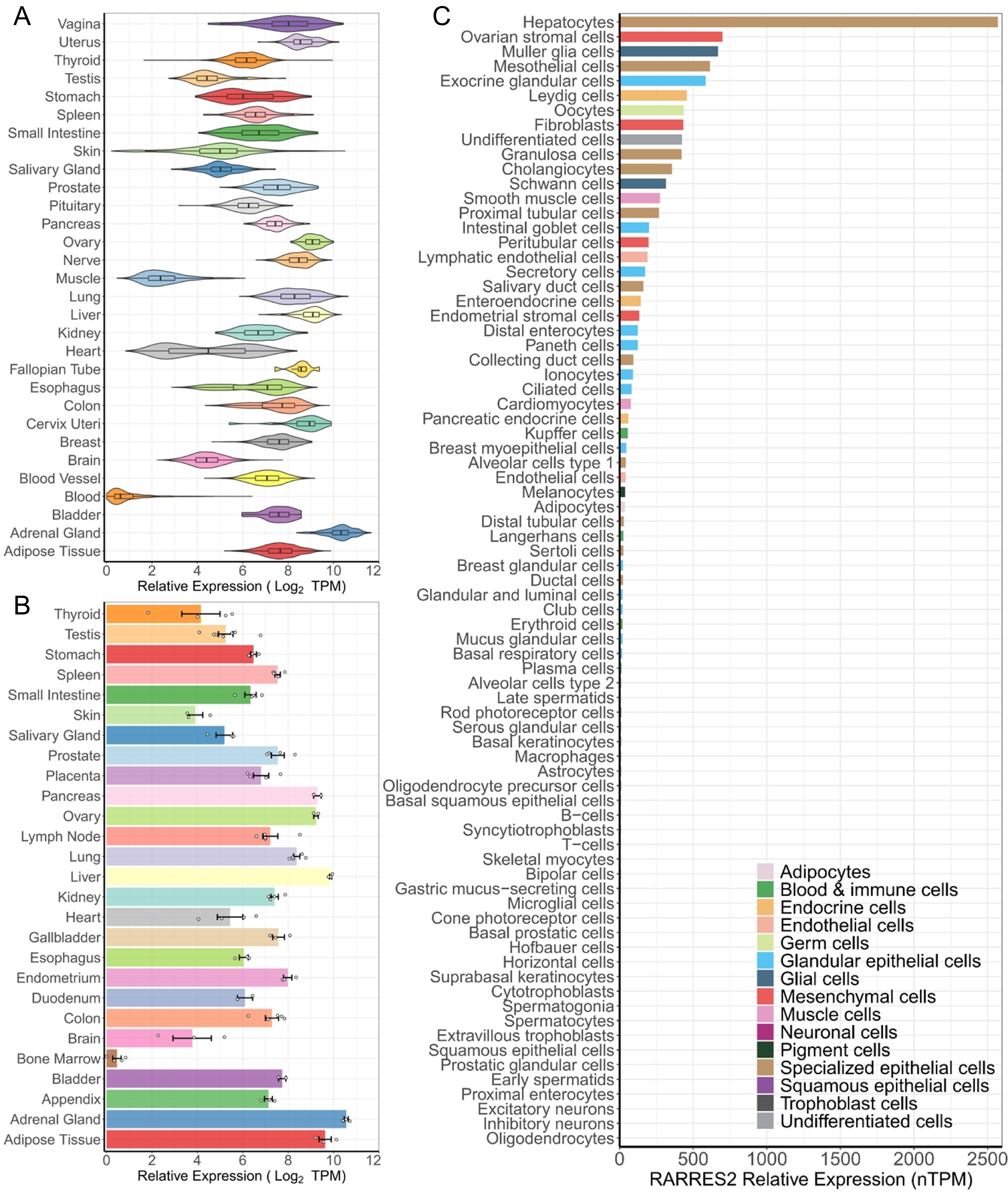

Supplement: S10 Fig — (C) RARRES2 expression distribution across normal cell types in the HPA database. (PNG) [file pone.0349749.s010.png]

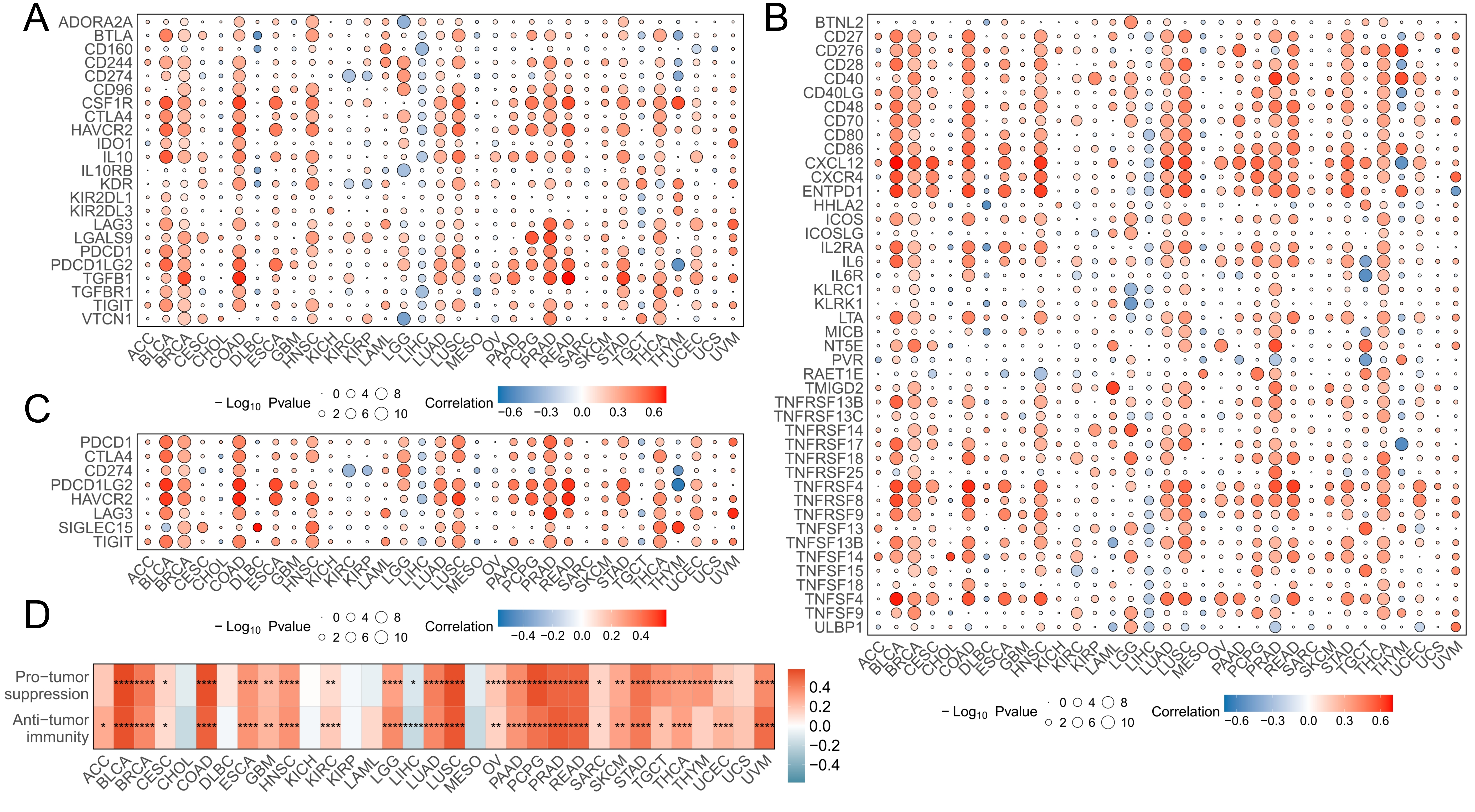

Supplement: S11 Fig — Dot plots depicting associations of RARRES2 with (A) immunoinhibitors, (B) immunostimulators, and (C) immune checkpoint genes. Circle size represents the P value, and color denotes the Spearman correlation coefficient. (D) Correlation heatmap showing associations of RARRES2 with infiltration of anti-tumor immune cells (ActCD4, ActCD8, TcmCD4, TcmCD8, TemCD4, TemCD8, Th1, Th17, ActDC, CD56briNK, NK, NKT) and pro-tumor immunosuppressive cells (Treg, Th2, CD56dimNK, imDC, TAM, MDSC, neutrophils, and pDC). (PNG) [file pone.0349749.s011.png]
